# Phytotoxicity, Cytotoxicity, and Antimicrobial Activity of Triethanolammonium Amino Acids Salts

**DOI:** 10.3390/molecules30081712

**Published:** 2025-04-11

**Authors:** Barbara Hanna Roman, Magdalena Charęza, Radosław Drozd, Martyna Sokołowska, Peter Sobolewski, Ewa Janus

**Affiliations:** 1 Department of Organic Chemical Technology and Polymer Materials, Faculty of Chemical Technology and Engineering, West Pomeranian University of Technology in Szczecin, Pułaskiego 10, 70-322 Szczecin, Poland; roman.barbara@zut.edu.pl; 2 Department of Microbiology and Biotechnology, Faculty of Biotechnology and Animal Husbandry, West Pomeranian University of Technology in Szczecin, Piastόw 45, 70-311 Szczecin, Poland; magdalena.szymanska@zut.edu.pl (M.C.); radoslaw.drozd@zut.edu.pl (R.D.); 3 Department of Polymer and Biomaterials Engineering, Faculty of Chemical Technology and Engineering, West Pomeranian University of Technology in Szczecin, Piastόw 45, 70-311 Szczecin, Poland; martyna.sokolowska@zut.edu.pl (M.S.);

**Keywords:** amino acids ionic liquids, phytotoxicity, cytotoxicity, antimicrobial activity

## Abstract

The growing use of ionic liquids (ILs) necessitates an understanding of their environmental impact and toxicity levels. In this study, a series of amino acid-based ionic liquids containing the triethanolammonium (TEA) cation were evaluated for their biological activity against *Lepidium sativum* L., the mouse fibroblast cell line L929, a selection of gram-positive and gram-negative bacteria, and the yeast *Candida albicans*. The influence of amino acid anion structure on toxicity was also examined. Among the tested ionic liquids, [TEA][Asp] exhibited low toxicity toward *Lepidium sativum L.*, representing terrestrial plants, while [TEA][Phe] showed the lowest cytotoxicity. Regarding microbial activity, [TEA][Lys] demonstrated greater bactericidal effectiveness against *E. coli* than *S. aureus*, while both [TEA][Lys] and [TEA][Arg] exhibited the strongest inhibitory effect against *C. albicans.* Our findings underscore the crucial role of IL salt composition in determining biological activity, highlighting the significance of interactions between IL components in shaping their potential effects.

## 1. Introduction

Ionic liquids (ILs) constitute an exceptionally numerous and intensively studied group of chemical compounds. The unique properties of ILs result from several aspects, including the characteristic salt structure, consisting of an organic cation and an inorganic or organic anion, which primarily determine their liquid state at temperatures below 100 °C. As a rule, many of them are liquid at room temperature and occur in the liquid state in a wide range of temperatures. The relatively low melting point temperatures of these salts are closely related to their specific crystal lattice, created by anions and cations of different size and shape, often with a dispersed charge [1].

Ionic liquids have high application potential and are of interest in various fields of science and industry. Negligible vapor pressure, non-flammability, and various miscibilities with water or thermal stability are just some of the beneficial properties that make ILs attractive for use in the extraction of active compounds from plants [2], in biomass conversion [3], in organic synthesis [4] as reaction media [5] or catalysts [4,6], in electrochemistry [3,7], or in medicine and pharmacology [8]. Ionic liquids can be modified by introducing various cations and anions and functional groups into their structure, which creates a wide range of possibilities in the field of designing structures with favorable physical and chemical properties. Their use can contribute to the implementation of more environmentally sustainable and efficient processes that will also be economically viable. The wide usefulness of these substances also requires multidirectional studies assessing the impact on the environment and the degree of their ecotoxicity. Concerns about the environmental risk of ILs have been raised due to their potential to enter the environment through accidental spills and industrial effluents. Various bioindicators, from enzymes through cell cultures, microorganisms, aquatic and terrestrial plants to vertebrates, have been used [9,10,11] to better understand their adverse effect on the organisms of various levels of organization and from varied trophic levels. In addition to the beneficial effect resulting in the destruction of harmful organisms, such as pathogenic bacteria, fungi, or weeds, they may also disturb the role the organisms play in the balance of ecosystems. Toxicity of ILs is dependent on IL concentration [12], type of the cationic core [13], length of the side chain in the cation [14,15], and the nature of the anion [9,15]. Furthermore, toxicity may vary for different organisms [16]. The studies of acute toxicity of ionic liquids with various cationic heads towards three representative freshwater organisms demonstrated that toxicity decreases on going from aromatic heterocyclic nitrogen-containing cation to non-aromatic cyclic and acyclic head. Moreover, the substitution of one or two carbon atoms in the alkyl chain with a more electronegative atom (chlorine or oxygen) reduced the acute toxicity. The substitution of an alkyl chain with a hydrogen atom reduced toxicity, too [16,17,18,19]. This knowledge allows the design of new ionic liquids with lower toxicity to overcome the threat they pose to the environment. The particular attention in the choice of the anion or cation of ILs is directed to biocompatible compounds such as amino acids (AA).

Baharuddin et al. studied the ecotoxicity of six amino acids ILs against zebrafish. The selected cations were tetrabutylphosphonium [P_4444_], tetrabutylammonium [N_4444_], and cholinium [Cho], and the anions chosen were the phenylalaninate [Phe] and taurinate [Tau]. The acute toxicity studies indicated that all ILs had LC50 values after 96 h higher than 100 mg L^−1^ and would be classified as practically harmless [20].

The toxicity assessment of 1-(2-hydroxyethyl)-3-methylimidazolium [C_2OH_MIM] salts of four amino acids—proline, glycine, serine, and alanine—towards aquatic organisms *Scenedesmus quadricauda* (green algae) and *Vibrio fischeri* revealed that these salts are non-toxic. The EC_50_ values were between 3.14 and 4.58 mg L^−1^ for algae and between 8.12 and 14.51 mg L^−1^ for *V. fischeri*, but the highest EC_50_ and the least toxicity for both organisms provided the prolinate anion. According to the authors, the non-toxicity was due to the short hydroxyl-functionalized alkyl group in the cation and the environmentally friendly nature of the amino acid anions [21].

In other studies, the amino acid anions were combined with the choline cation, considered to be relatively non-toxic and environmentally friendly [22,23]. The toxicity of cholinium serinate [Cho][Ser], cholinium valinate [Cho][Val], cholinium prolinate [Cho][Pro], cholinium histidinate [Cho][His], and cholinium alaninate [Cho][Ala] towards guppy fish (*Poecilia reticulate*) and three microbial species—*Listeria monocytogenes*, *Aeromonas hydrophila,* and *Klebsiella pneumoniae*—exhibited that the studied ILs could be classified as non-toxic and their antimicrobial potency was lacking to fully function as an antibiotic. The antimicrobial activity of ILs depended on the concentration and time of application. The reported EC_50_ values for the same microbial species were practically comparable for all tested cholinium ILs and even higher than for the earlier reported [C_2OH_MIM] analogues. The toxicity data indicated no clear trend for the effect of the different amino acid anions; that depended on the test organism. [Cho][Ser] was characterized by the lowest antimicrobial activity against two gram-negative bacteria—*K. pneumoniae* and *A. hydrophila*—while the highest activity was exhibited by [Cho][Ala] and [Cho][Pro], respectively. However, among all tested amino acid cholinium salts, [Cho][Ser] presented the highest antimicrobial activity against gram-positive *L. monocytogenes*. Moreover, based on EC_50_ values, [Cho][AA] salts were shown to be ten times less toxic than the respective 1-methyl-3-octylimidazolium amino acids salts, [OMIM][AA] [21,24]. For the ionic liquids composed of 1-methyl-3-octylimidazolium cation and various anions, a linear quantitative structure-antibacterial activity relationship (QSAR) model was used. The anion effect was evaluated against four bacteria: *L. monocytogenes*, *S. aureus*, *E. coli,* and *A. hydrophila,* and the toxicity decreased in the following order: [N(CN)_2_] > [Br] > [BF_4_] > [Cl] > [Asp] > [Gly] > [Ala] > [Pro] > [Ser] for all species [25]. Thus, amino acid anions are responsible for the significantly lower toxic effect of ILs. The lowest antimicrobial activity of ILs with the L-serinate anion is attributed to the hydroxyl group in the side chain of L-serine. Similarly, the lower toxicity of salts with the choline or 1-(2-hydroxyethyl)-3-methylimidazolium cation is assigned to the functionalization of the alkyl substituent in the cation with the hydroxyl group.

**Scheme 1:**
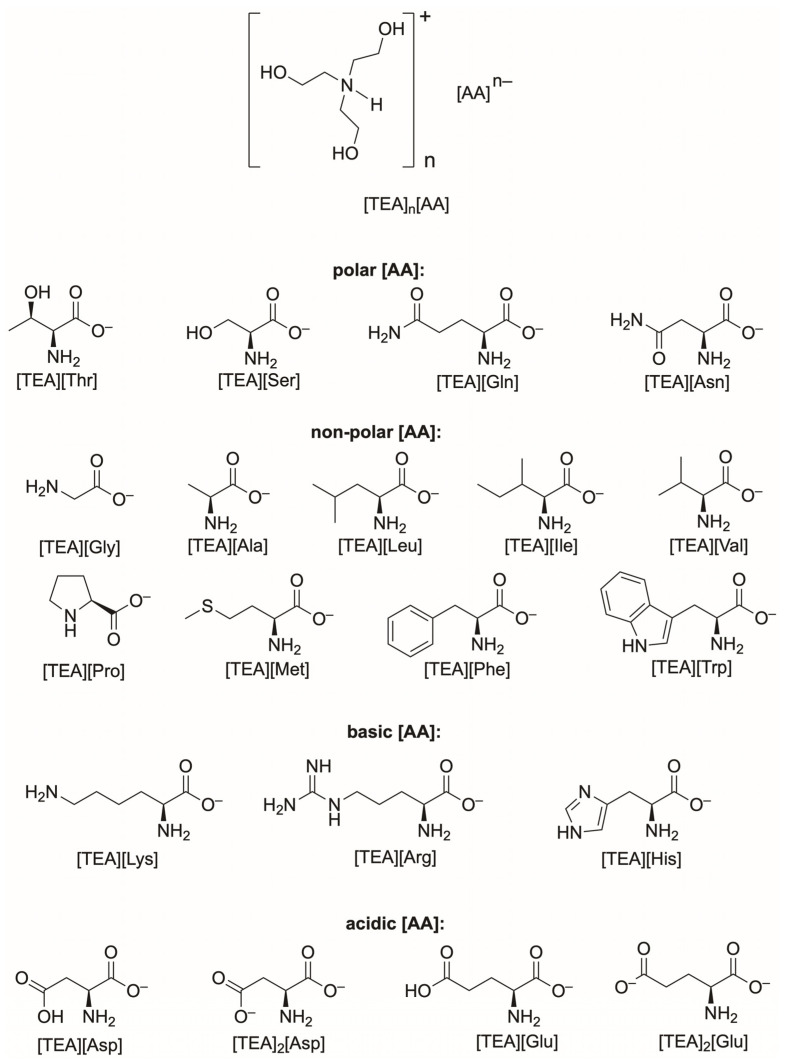
Structure of triethanolammonium salts of various amino acids.

Considering the above literature reports demonstrating the reduced toxicity of ionic liquids by functionalization of the cation with hydroxyl groups and using amino acids as anion sources we have recently obtained triethanolammonium salts of various amino acids [TEA][AA] (Scheme 1). In our previous article, we described their physicochemical properties and the effect on the activity of selected proteases [26]. In this paper we develop studies on the toxicity of TEA amino acid salts towards *Lepidium sativum* L., mouse fibroblast cells line L929, yeast, and gram-positive and gram-negative bacteria. The presented results enhance the knowledge about either the safety of their use or the environmental hazard. The use of three distinct biological models was not only ecotoxicologically justified but also motivated by the search for potential applications of these compounds—whether in agriculture (as low toxicity plant biostimulants), biotechnology (as selective antimicrobials), or pharmaceutical contexts (as biocompatible excipients). This exploratory strategy dictated a broader and more diverse biological screening approach.

## 2. Results and Discussion

### 2.1. Phytotoxicity of [TEA][AA] Salts 

Phytotoxicity assessment was carried out with cress seeds (*Lepidium sativum* L.) as an example species of terrestrial plants. *Lepidium sativum* L. is an excellent bioindicator due to its rapid growth and high sensitivity [27]. The test is analogous to the standard ISO 18763:2016 [28]. The effect of the type and concentration of [TEA][AA] salts were assessed in two bioassays. In the first, the degree of cress seeds germination, and in the second—the root growth in pregerminated cress seed, at 24 h after the beginning of incubation in the presence of salt solution—were measured. Inhibition of seed germination (%I_SG_) and root growth (%I_RG_) relative to a control sample were determined. The %I_SG_ and %I_RG_ values are listed in the Supplementary Materials in Tables S1 and S2, respectively. In addition, photos of Petri dishes showing the effect of [TEA][AA] salt concentration on the degree of cress seed germination (Figures S1–S3) and root growth (Figures S4–S6) are included in the Supplementary Materials.

**Figure 1:**
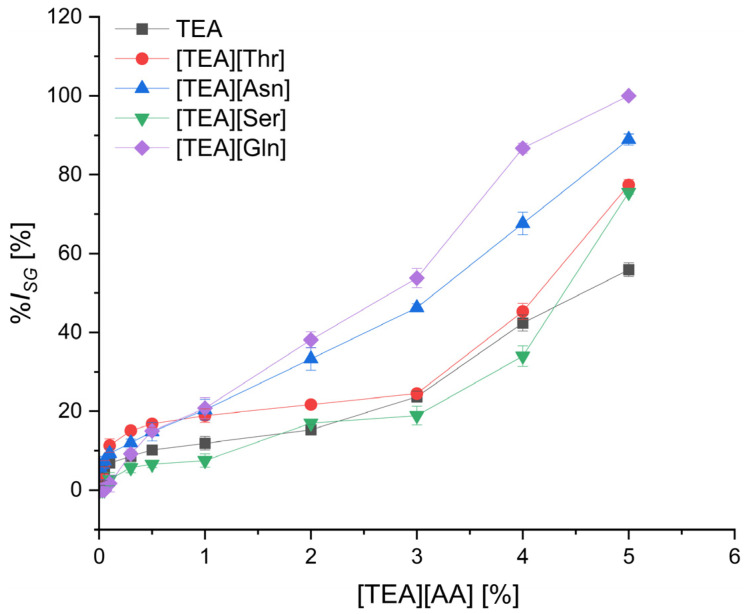
The inhibition level of cress seed germination (%I_SG_) by TEA salts with polar amino acids depending on their concentration; mean value of three replicates, error bars sign a standard deviation.

**Figure 2:**
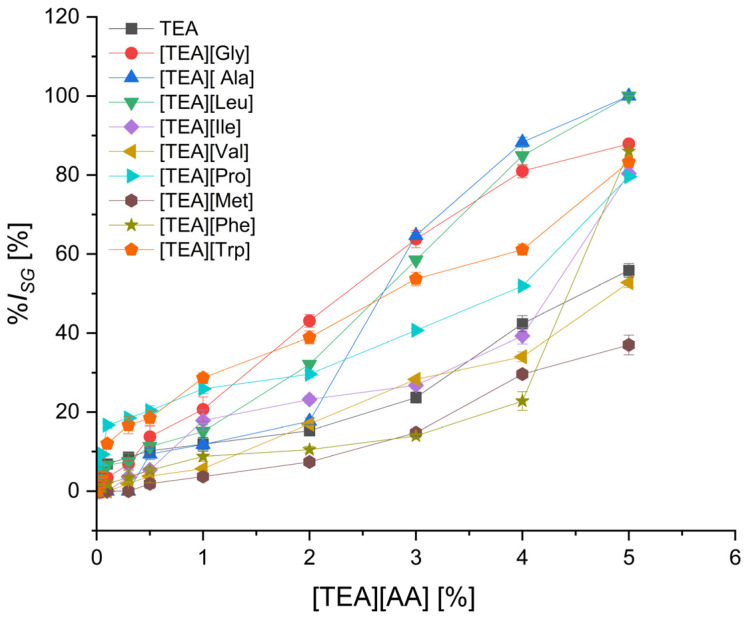
The inhibition level of cress seed germination (%I_SG_) by TEA salts with non-polar amino acids depending on their concentration; mean value of three replicates, error bars sign a standard deviation.

**Figure 3:**
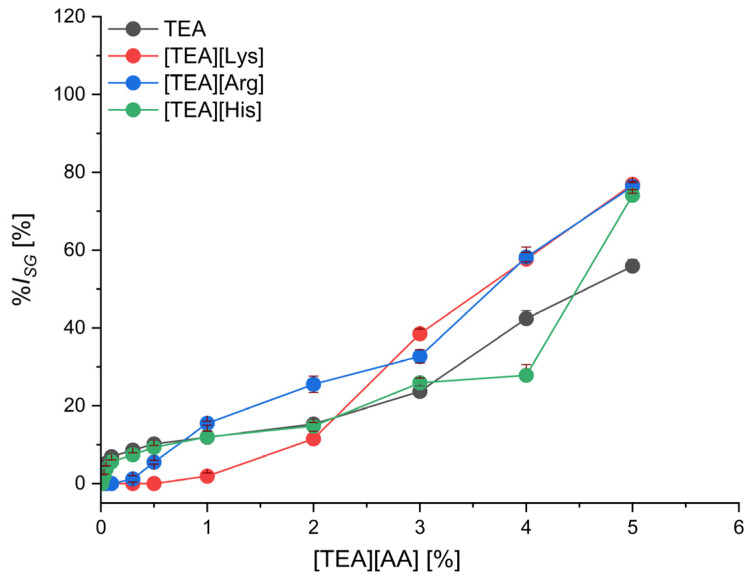
The inhibition level of cress seed germination (%I_SG_) by TEA salts with basic amino acids depending on their concentration; mean value of three replicates, error bars sign a standard deviation.

**Figure 4:**
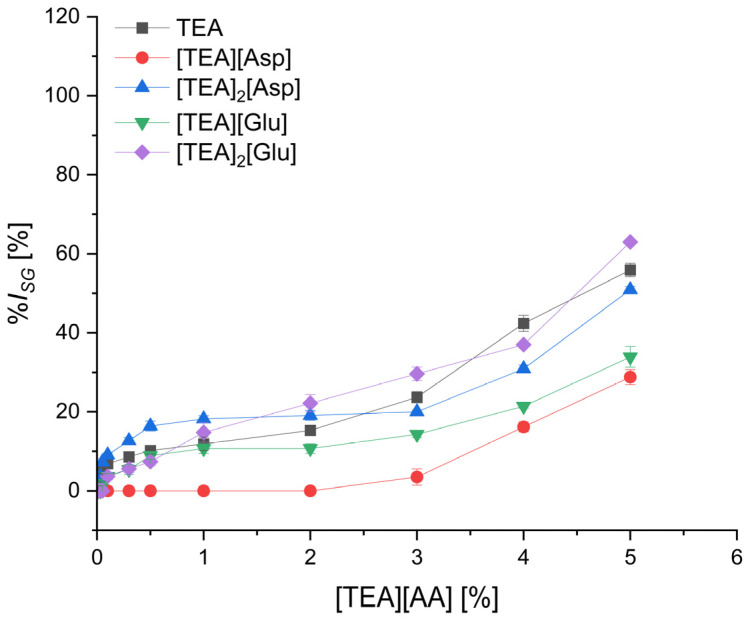
The inhibition level of cress seed germination (%I_SG_) by TEA salts with acidic amino acids depending on their concentration; mean value of three replicates, error bars sign a standard deviation.

The effect of [TEA][AA] salts concentration in the range from 0.01% to 5.0% on the decrease of the cress seeds germination in comparison to the control containing only water in the substrate was assessed. The dependence of seed germination inhibition (%I_SG_) on the concentration of the compound is presented in Figure 1, Figure 2, Figure 3 and Figure 4. Data are grouped into four categories based on the type of amino acid—polar (Figure 1), nonpolar (Figure 2), basic (Figure 3), and acidic (Figure 4) in the salt. Data for starting triethanolamine, TEA, are added to each graph.

In the group of polar amino acids (Figure 1), seed germination was inhibited to the lowest extent for the salts with anions of serine [TEA][Ser] and threonine [TEA][Thr], containing a hydroxyl group in the side chain, and was comparable to that for TEA. A little increase in %I_SG_, up to about 20%, was observed with an increase in salts concentration to 3.0%. Further increase in concentration above 3.0% caused a stronger inhibition of germination, and finally for the highest concentration of 5.0%, the %I_SG_ was over 70%, both for [TEA][Ser] and [TEA][Thr]. In contrast, for [TEA][Asn] and [TEA][Gln], containing an amide group in the side chain, an increase in germination inhibition was observed with increasing concentration, and at a concentration of 3.0%, the %I_SG_ was 2 to 2.5 times higher than for the [TEA][Ser] and [TEA][Thr] salts. Moreover, when [TEA][Gln] was used at a concentration of 5.0%, the germination was completely inhibited.

In the group of nonpolar amino acid salts (Figure 2), the seed germination was inhibited at the least degree for [TEA][Met] and [TEA][Phe], practically in the entire concentration range, except for the highest concentration of 5.0% for [TEA][Phe]. For [TEA][Met] and [TEA][Phe] used at a concentration of 3.0%, the %I_SG_ was lower than 15%, which is also lower compared to the least phytotoxic salts of polar amino acids—[TEA][Ser] and [TEA][Thr]. The highest germination inhibition among nonpolar amino acid salts was observed for [TEA][Trp], as well as [TEA][Gly] and [TEA][Leu]—for a concentration of 3.0%, the seed germination inhibition was 53.7%, 63.8%, and 58.5%, respectively. For the highest concentration of 5.0% of these salts, the germination was inhibited by 90% to 100%.

In the group of basic amino acid salts (Figure 3), the seed germination inhibition for each amino acid is similar and comparable with TEA. Lysine salt can be considered the least toxic at concentrations up to 2.0%, as it inhibits seed germination by 11.5%. For concentrations higher than 2.0%, arginine and lysine salts showed the highest phytotoxicity. Germination inhibition at 3.0% concentration was 32.7% and 38.5% for [TEA][Arg] and [TEA][Lys], respectively, and above 76% for both salts used at 5.0% concentration.

The group of salts based on acidic amino acids (Figure 4) ensured the lowest level of germination inhibition. The lowest germination inhibition was found for monocationic salts of aspartic acid, [TEA][Asp], and glutamic acid, [TEA][Glu]—at a concentration of 3%, the %I_SG_ was 3.5% and 14.3%, respectively, and at a concentration of 5.0%–28.8% and 33.9%, respectively. The bicationic salts [TEA]_2_[Asp] and [TEA]_2_[Glu] inhibited seed germination to a greater degree than monocationic and similarly to the starting TEA (%I_SG_ = 55.9%) and salts of polar amino acids Ser and Thr.

The inhibition increased with the increase in [TEA][AA] salts concentration. Moreover, the seed coat became darker and darker with the higher concentration of salts (Figures S1–S3 in Supplementary Materials). The darker color may be the result of the triethanolammonium cation connection to the surface of the seed coat or to the secreted exudate lepidimoid (sodium 2-OL-rhamnopyranosyl-4-deoxy-alpha-L-threo-hex-4-ene-pyranoside duronate) in the germinated cress seeds.

**Figure 5:**
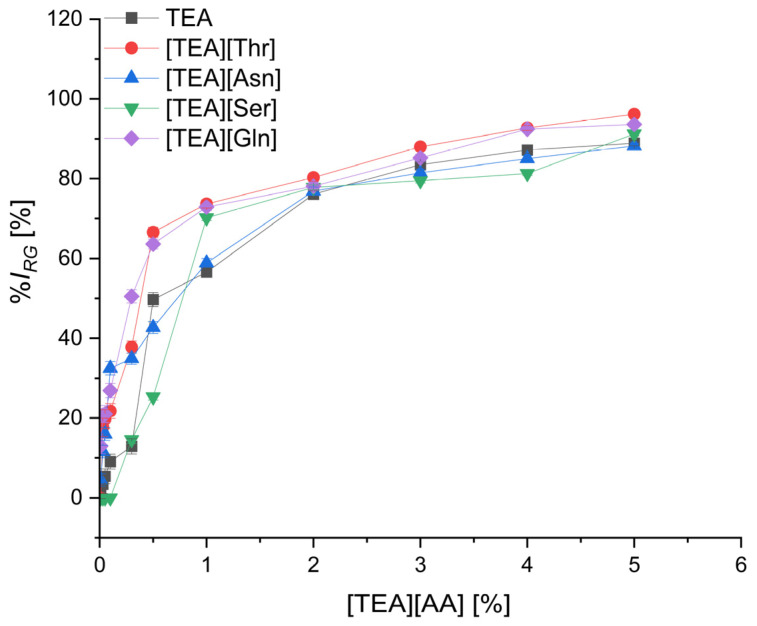
The inhibition level of cress root growth (%I_RG_) by TEA salts with polar amino acids depending on their concentration; mean value of three replicates, error bars sign a standard deviation.

**Figure 6:**
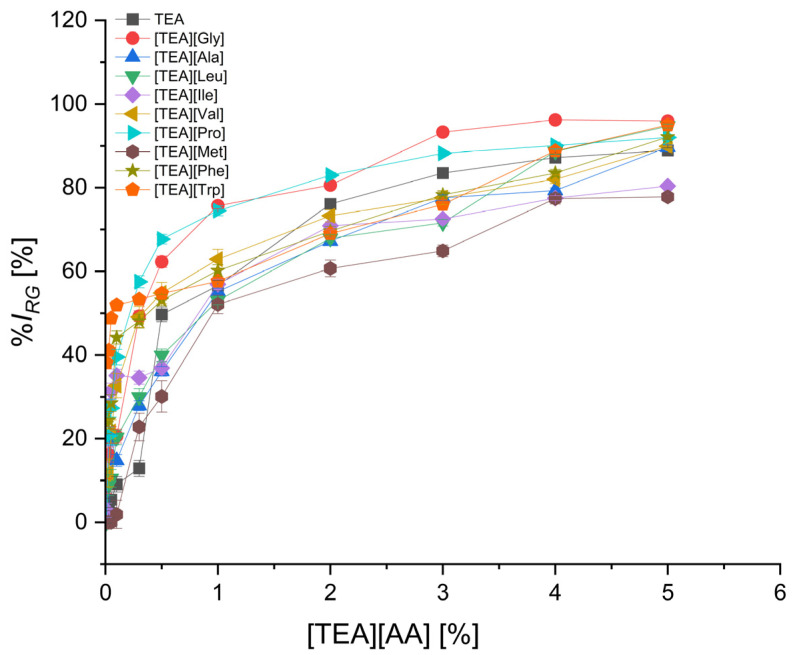
The inhibition level of cress root growth (%I_RG_) by TEA salts with non-polar amino acids depending on their concentration; mean value of three replicates, error bars sign a standard deviation.

**Figure 7:**
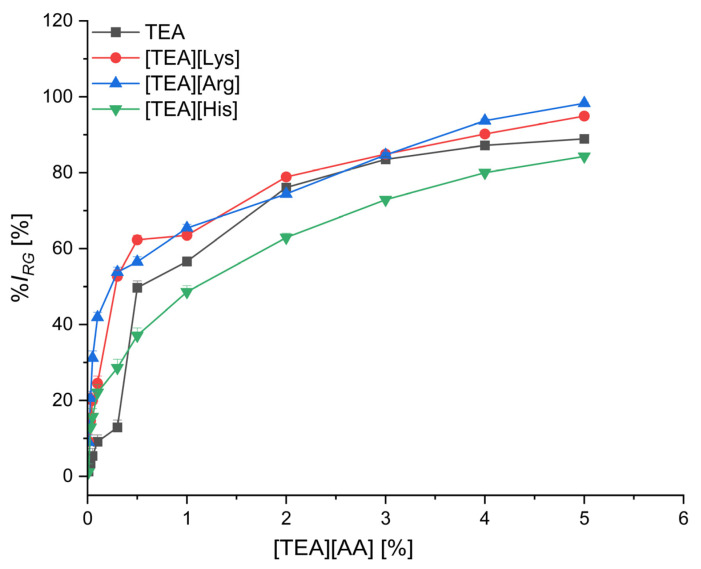
The inhibition level of cress root growth (%I_RG_) by TEA salts with basic amino acids depending on their concentration; mean value of three replicates, error bars sign a standard deviation.

**Figure 8:**
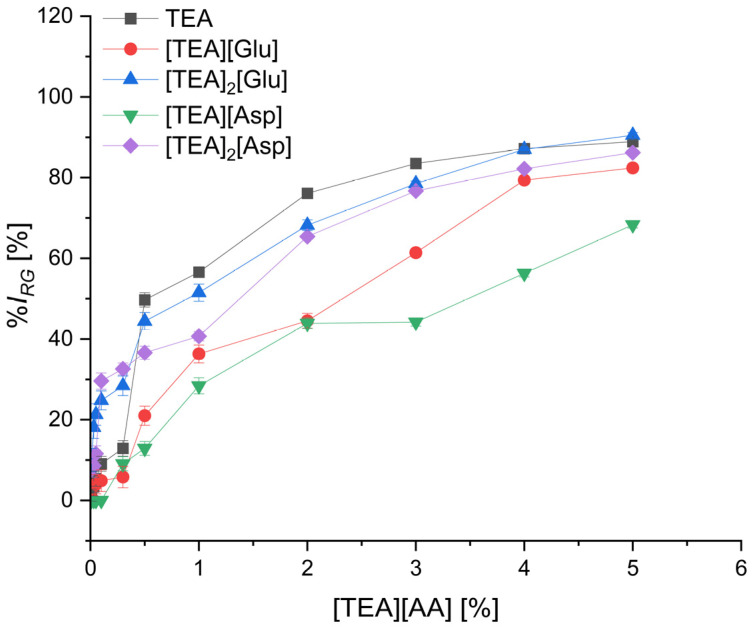
The inhibition level of cress root growth (%I_RG_) by TEA salts with acidic amino acids depending on their concentration; mean value of three replicates, error bars sign a standard deviation.

The second phytotoxicity test concerned the effect of [TEA][AA] salts and their concentration in the range of 0.01–5.00% on the inhibition level of root growth (%I_RG_), as shown in Figure 5, Figure 6, Figure 7 and Figure 8, respectively, for polar (Figure 5), non-polar (Figure 6), basic (Figure 7), and acidic (Figure 8) amino acids comprising the salt.

Dependence of root growth inhibition level on salt concentration has a similar shape for each salt (Figure 5, Figure 6, Figure 7 and Figure 8). An increase in concentration up to about 1.0% causes a significant, even 80%, inhibition level of root growth, and further increases in concentration cause only slight changes in inhibition level; practically the curves reach a plateau for concentrations higher than 1.0%. The shape of this curve is different than for the effect of salt concentration on the inhibition level of germination (Figure 1, Figure 2, Figure 3 and Figure 4), where the increase in concentration, usually up to 3.0%, caused a slight inhibition of germination, and concentrations higher than 3.0% resulted in a sharper increase in inhibition. Probably, the process of seed germination is dependent to a lesser extent on the substrate composition because during germination the materials accumulated in the seeds are used.

In the root growth inhibition bioassay, a twisting of roots was observed for concentrations of [TEA][AA] salts above 0.5%. Root twisting can be the effect of a change in osmotic pressure in the presence of [TEA][AA] salts. The probable effect of [TEA][AA] salts on the root growth is related to the inhibition of mitotic divisions, the inhibition of the activity of hydrolytic enzymes, and the slowdown of cell elongation growth (Figures S4–S6 in Supplementary Materials).

In the group of polar amino acids (Figure 5), the root growth was inhibited to the lowest level by salt of serine [TEA][Ser], which contains a hydroxyl group in the side chain. Practically, up to a concentration of 0.1% [TEA][Ser], no inhibition compared to the control was noted. A small increase in %I_RG_ to 14.6% was observed with an increase in [TEA][Ser] concentration to 0.3%. Comparable inhibition of root growth (%I_RG_ = 12.9%) was obtained for the same concentration of TEA. However, the salts of other polar amino acids [TEA][Asn], [TEA][Thr], and [TEA][Gln], used in a concentration of 0.3%, inhibited statistically significant root growth at the level of 34.9%, 37.8%, and 50.5%, respectively (Table S5).

In the group of nonpolar amino acid salts (Figure 6), the least inhibition of root growth, lower than for the starting TEA, was observed for the [TEA][Met] salt, practically in the entire concentration range. For [TEA][Met] used in a concentration of 0.1%, the %I_RG_ was less than 2%. The highest inhibition of root growth in the group of nonpolar amino acid salts was observed for [TEA][Trp] and [TEA][Phe] salts as well as [TEA][Val] and [TEA][Gly] (Table S7). Even at the lowest applied concentration of 0.01%, the inhibition of root growth was 38.1%, 23.6%, 11.6%, and 8.7%, respectively. These four salts applied at the highest concentration of 5.0% inhibited the root length growth by 90–96%.

In the group of basic amino acid salts (Figure 7 & Table S6), [TEA][His] could be considered as less phytotoxic in comparison to [TEA][Lys] and [TEA][Arg] salts. For example, at a concentration of 0.3%, the inhibition of root growth by [TEA][His] was 28.6% and was 2-fold lower than for [TEA][Lys] and [TEA][Arg] salts.

TEA salts of acidic amino acids (Figure 8) were characterized by the least inhibition of the cress root growth. Monocationic salts of the aspartic acid [TEA][Asp] as well as glutamic acid [TEA][Glu] used at the concentration of 0.3% inhibited the root growth by only 9.1% and 5.8%, respectively. In the case of bicationic salts of these two amino acids, the inhibition of root growth was significantly higher (Table S4) and comparable to TEA. The inhibition increased with the increase in the concentration of [TEA][AA], but even for the highest concentration of 5.0%, the lowest root growth inhibition was for [TEA][Asp] (%I_RG_ = 68.3%).

The salts [TEA][Asp], [TEA][Glu], and [TEA][Ser] can be considered as the least phytotoxic at the concentration up to and including 0.5%. Root growth inhibition using these salts at a concentration of 0.5% was 12.9%, 21%, and 25.3%, respectively. Moreover, these three salts are less toxic than TEA, for which %I_RG_ was 49.7%, at a concentration of 0.5%. The most toxic effect on root growth causes [TEA][Trp], which, applied at the lowest concentration of 0.01%, inhibited root growth by 38.1%. An equally high level of toxicity was characterized by [TEA][Phe] with %I_RG_ on the level of 23.6% at the same concentration of 0.01% (Tables S4 and S5).

Considering the phytotoxicity of [TEA][Ser] and [TEA][Thr], the higher toxicity of [TEA][Thr] can be the effect of the longer alkyl chain and different position of the hydroxyl group in the side chain of threonine. A similar tendency was observed comparing [TEA][Asn] and [TEA][Gln], where in the case of the latter salt, the longer alkyl chain resulted in greater inhibition. Comparing three salts, [TEA][His], [TEA][Lys], and [TEA][Arg], the highest inhibition was found for [TEA][Arg], both in the seed germination test and the root growth test. In the case of triethanolammonium salts of acidic amino acids, dicationic salts [TEA]_2_[Asp] and [TEA]_2_[Glu] showed a higher inhibition of seed germination and root growth than the monocationic salts [TEA][Asp] and [TEA][Glu]. This can be attributed to the higher proportion of TEA in the dicationic salts.

Based on two bioassays—cress seed germination and root growth—it can be concluded that the combination of TEA with the amino acids Asp, Met, and Ser provided the lowest phytotoxicity. 

Considering the series of [TEA][AA] according to the no observed effects concentration (NOEC) for cress seed germination (Table 1) and cress root growth (Table 2), [TEA][Asp] salt was characterized by the lowest phytotoxicity. On this basis, it can be concluded that aspartic acid significantly eliminates the phytotoxic properties of the cation. The other salts can be ranked according to increasing NOEC value for both tests and decreasing toxicity as follows: [TEA][Met], [TEA][Ala], and [TEA][Ser].

In our studies of the [TEA][AA] salts effect on the cress seed germination and root growth, we used higher salt concentrations than in studies presented by other authors in the same bioassays. For example, compared to concentrations between 0.0003 and 0.03 mg L^−1^ applied by Kondratenko et al. [12], the concentrations of [TEA][AA] salts in the range from 100 mg L^−1^ to 50,000 mg L^−1^ used by us were about a million times higher. Kondratenko et al. studies [12] revealed that TEA salts of benzoic, salicylic, cinnamic, oxalic, succinic, malonic, malic, and citric acid in all tested concentrations were characterized by the lack of a statistically significant effect on the cress growth. However, for TEA salicylate used at the highest concentration of 0.03 mg L^−1^ and TEA citrate at a concentration of 0.0003 mg L^−1^ a significant inhibition effect on root and shoot length was determined by authors. Moreover, it was found that TEA salts of cinnamic, benzoic, and malonic acids had a positive stimulating effect on the seed germination and root growth of cress. The most visible stimulation of cress seed germination and root growth was shown by TEA salt of cinnamic acid at concentrations of 0.003 and 0.03 mg L^−1^. It can be expected that [TEA][AA] salts used by us in such low concentrations as by Kondratenko could also show a stimulating effect on cress, due to comprising the amino acids, which play important roles in plant growth and stress control and are effective biostimulants for plants. Our achievement is to demonstrate that even at concentrations as high as 0.01% to 2% (corresponding to 100–20,000 mg L^−1^), there are [TEA][AA] salts that do not cause a negative effect on the germination and growth of cress roots.

Our results can also be compared to the studies conducted by Studzińska and Buszewski [17] that concerned the effect of imidazolium ionic liquids ([EMIM][Cl], [BMIM][Cl], and [HMIM][Cl]) in the concentration range of 0.01–1000 mg L^−1^, on the cress seed germination. The authors reported that concentrations from 0.01 to 0.1 mg L^−1^ of all ILs did not affect root growth and were considered safe. However, at the highest concentration of 1000 mg L^−1^ for [BMIM][Cl] and [HMIM][Cl], the decrease in germination was 100%, and for [EMIM][Cl], it was 85%. In relation to these studies [17], [TEA][AA] salts tested by us showed no germination inhibition at the same concentration of 1000 mg L^−1^ (0.1%) and even at higher concentrations, in the range between 3000 and 20,000 mg L^−1^ (0.3–2.0%) (Table 1). Our results confirm no phytotoxic effect of TEA amino acid salts compared to imidazolium ionic liquids [17].

### 2.2. Mammalian Cell Cytotoxicity of [TEA][AA] Salts 

To evaluate cytotoxic or growth-inhibitory effects of [TEA][AA] salts, an in vitro assay was performed using mouse L929 fibroblasts. Screening experiments were performed at two different exposure times (24 h and 48 h) over a concentration range of <0.001% to 5%. The obtained results regarding the cytotoxic effect of amino acid ionic liquids on the mouse fibroblast cell line (L929) are presented in Supplementary Materials (Table S3). The cytotoxicity test was performed using the National Cancer Institute 60 panel [29] for 24 and 48 h. A four-parameter dose-response model (logistic or Weibull) was used to calculate 50% effective doses (ED_50_). The doubling time of L929 is typically around 20–22 h, so viability values below 50% indicate cytotoxicity.

**Figure 9:**
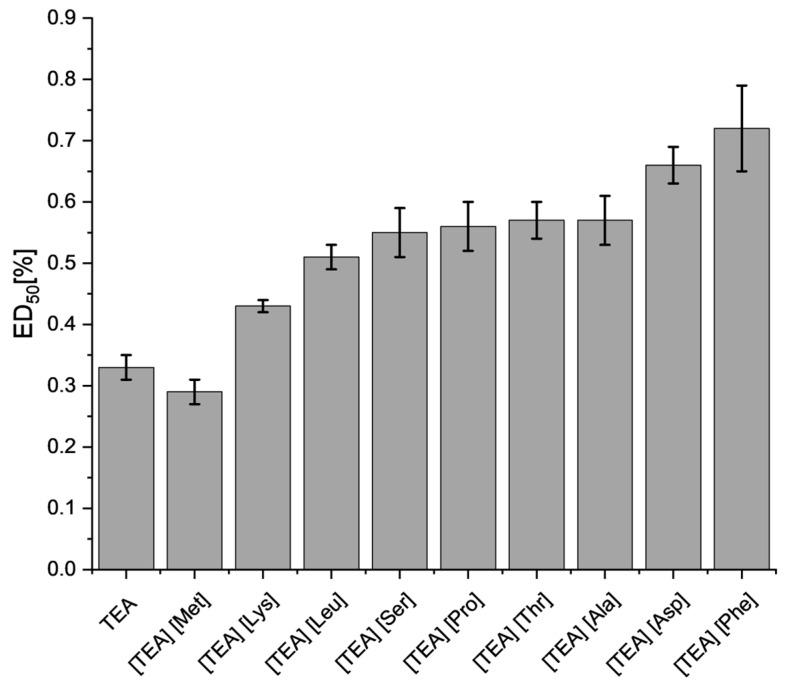
The ED_50_ values for L929 fibroblasts after 24 h exposure to [TEA][AA] salts. The error bars indicate a standard error of the mean value.

After 24 h of culture, all tested compounds were cytotoxic at concentrations > 1%; however, the most negative effect on the viability of L929 cells was observed after exposure to [TEA][Met], whose ED_50_ was 0.29% ± 0.02 (Figure 9). The second compound causing acute cell cytotoxicity was TEA, for which the ED_50_ was 0.33% ± 0.02. Interestingly, [TEA][Phe] with an aromatic side chain was characterized by the lowest degree of cytotoxicity (ED_50_ = 0.72% ± 0.07) compared to TEA. The use of an amino acid as an anion in the design of an ionic liquid reduces the cytotoxic effect of TEA. [TEA][AA] salts with the acidic anion [TEA][Asp] were less toxic than the basic [TEA][Lys]. Similar ED_50_ values were observed for [TEA][Leu], [TEA][Pro], [TEA][Ser], [TEA][Thr], and [TEA][Ala].

The ED_50_ values for [TEA][AA] salts are ranked by decreasing cytotoxicity after 24 h:

[TEA][Met] > TEA > [TEA][Lys] > [TEA][Leu] > [TEA][Ser] > [TEA][Pro] > [TEA][Thr] ≈ [TEA][Ala] > [TEA][Asp] > [TEA][Phe]. 

**Figure 10:**
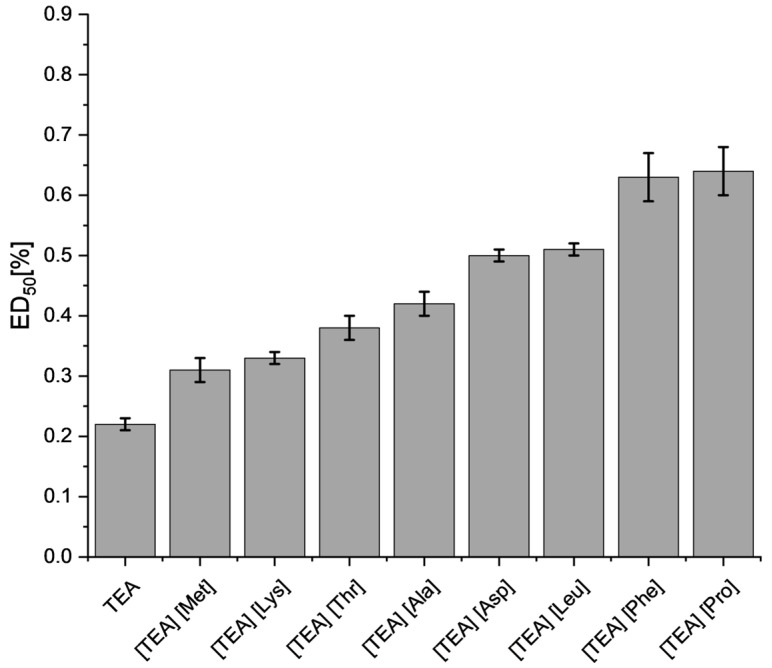
The ED_50_ values for L929 fibroblasts after 48 h exposure to [TEA][AA] salts. The error bars indicate a standard error of the mean value.

While an increase in cytotoxicity of [TEA][AA] salts on L929 fibroblasts was observed after 48 h of exposure (Figure 10), the ED_50_ values (with few exceptions) are similar to those after 24 h (Figure 9), which indicates that the majority of the toxic effect was acute. TEA (0.22% ± 0.01) was characterized by the highest cytotoxicity in ED_50_. Only in the case of [TEA][Met] and [TEA][Pro], the ED_50_ cytotoxic effect slightly decreased, while in [TEA][Leu] it remained at the same level. A strong toxic effect after 48 h exposure to [TEA][AA] salts, which were ranked in descending toxicity order: 

TEA > [TEA][Met] > [TEA][Lys] > [TEA][Thr] > [TEA][Ala] > [TEA][Asp] > [TEA][Leu] > [TEA][Phe] > [TEA][Pro]. 

In the cell suspension, we also observed fluctuations in the pH of the medium, indicated by a pink (alkaline pH) or yellow (acidic pH) color change induced by the addition of TEA, [TEA][Lys], or [TEA][Asp], which has a negative effect on animal cells that are sensitive to pH changes. Therefore, such high cytotoxicity towards animal cells results not only from the properties of the above compounds but also from the effect of their pH. In the tests of the remaining compounds, there was no change in the color of the culture medium; the pH did not change. However, the combination of TEA with an amino acid anion reduced the cytotoxic effect compared to pure TEA. The change in cell viability depends on the concentration of ILs used as well as its structure. An equally frequently used cation is the choline cation, which belongs to the quaternary ammonium compounds known as cationic surfactants [30,31], which may justify the effect on reducing cell viability. However, choline ILs show less cytotoxicity compared to the more common imidazolium ILs. It is worth paying attention to the possibility of exerting a toxic effect on cells not only by the cation but also depending on the type of anion used in ILs. An increase in cytotoxicity was observed with the extension of the side chain in the amino acid itself [32,33]. Therefore, our team attempted to assess the toxicity of [TEA][AA] salts based on examples described in the literature. In the case of choline ILs with Gly and Ala anion, they induced a slight decrease in 3T3 cell viability (mouse embryonic fibroblasts) with increasing concentration over 48 h. Cell viability was over 90% at a concentration of about 0.5%, and the IC_50_ was about 55 mM (1%) for both ILs [34]. Almeida et al. found that for [Cho][Glu] and [Cho][Phe], the IC_50_ was 0.43% (*v*/*v*) and 0.42% (*v*/*v*), respectively [35]. With regard to their research, after 48 h of exposure of L929 cells in the case of [TEA][Ala], [TEA][Asp], [TEA][Leu], [TEA][Phe], and [TEA][Pro], ED_50_ values were obtained in the range of 0.42–0.64%, which coincides with the literature data and confirms the low toxicity of those [TEA][AA] salts. Only in the case of [TEA][Met], [TEA][Lys], and [TEA][Thr] were the ED_50_ values lower (0.31–0.38%), but still higher than the TEA reference substance. In addition, [Cho][Gly] and [Cho][Ala], when used in small amounts, increased the solubility of ibuprofen, and more importantly, did not increase cytotoxicity, so they can act as excipients [34]. Amino acid ionic liquids are less toxic than commonly used imidazolium and pyridinium ionic liquids [36]. The cytotoxicity of other ILs against murine L929 fibroblasts was also investigated. Agostinho et al. [37] determined the cytotoxicity profile for ionic liquids based on carboxylic anions and ammonium cations after 24 h exposure. Almost all ILs tested had an IC_50_ higher than 100 mM for L929 fibroblast cells. In addition, scientists emphasized that ILs containing dicarboxylate anion, the growth of the alkyl chain does not increase toxicity as is usually the case in monocarboxylate ILs. Silva et al. [38] studied the cytotoxic effect of a large set of dicationic ionic liquids on L929 mouse fibroblasts after 24 h and showed that the dicationic ionic liquids [N_12OH2OH2_C_6_][AcPhe_2_] and [N_12OH2OH2_C_6_][CH_3_CH_2_CH_2_CO_2_]_2_ are toxic. In this kind of IL, the anion was responsible for the toxicity. Moreover, it has been observed that dicationic ILs are much less toxic than monocationic ones. [N_12OH2OH2_C_4_][CH_3_CO_2_]_2_ and [N_12OH2OH2C2O_C_2_][CH_3_CO_2_]_2_ were the least toxic, and an increase in cytotoxicity was correlated to the increase in the length of the alkyl chain between the cations in ILs. In our studies, we came to a different conclusion, namely, depending on the amino acid anion used, a different degree of cytotoxicity is exerted. The triethanolammonium cation in combination with Met turned out to be the most toxic, and in combination with Phe, the least toxic after 24 h of exposure.

Egorova et al. [39] analyzed the influence of a series of ionic liquids based on imidazolium cations with amino acids and inorganic anions on the viability of two types of cell lines: NIH/3T3—mouse fibroblasts and CaCo-2—adenocarcinoma of the intestine and rectum. The ILs analyzed were less toxic to NIH/3T3 fibroblasts, except for [BMIM][BF_4_] and [HMIM][Cl], which showed similar toxicity to CaCo-2 and NIH/3T3. It was observed that with the increase in the length of the alkyl side chain in the cation ([EMIM][Cl], [BMIM][Cl], and [HMIM][Cl]), the toxic effect of ILs on cell lines increases. The influence of anion on the degree of toxicity was also observed, which was the lowest after the application of [BMIM][Ala] (IC_50_ = 19.35 mM), [BMIM][Cl] (IC_50_ = 18.85 mM), and [BMIM][(L)-Lac] (IC_50_ = 19.16 mM) against CaCo-2, while against NIH/3T3 they were [BMIM][Ala] (IC_50_ = 30.23 mM) and [BMIM][(L)-Lac] (IC_50_ = 33.65 mM). The highest degree of toxicity was determined for CaCo-2 in [BMIM][BF_4_] (IC_50_ = 11.19 mM) and [BMIM][PF_6_] (IC_50_ = 11.50 mM) and for NIH/3T3 in [BMIM][BF_4_] (IC_50_ = 11.30 mM). While Gly and Val anions were less toxic than BF_4_, PF_6_, and HSO_4_, they were more toxic than L-lactate anion. Also, the amino acid cations ([Gly-OMe][BF_4_], [Ala-OMe][BF_4_], and [Val-OMe][BF_4_]) showed comparable or higher toxicity compared to the other ILs [HMIM][Cl] and [BMIM][BF_4_]. In conclusion, the presence of an amino acid may not always result in lower IL toxicity, especially when an amino acid cation combined with a toxic anion, e.g., BF_4_^−^, may increase toxicity.

### 2.3. Antimicrobial Activity of [TEA][AA] Salts

The antimicrobial activities of a series of [TEA][AA] salts were tested using gram-positive *S. aureus*, gram-negative *E. coli*, and the yeast *C. albicans* as model microorganisms. The calculated average minimal inhibitory concentration (MIC) and minimal bactericidal concentration (MBC) values were listed and summarized in Table 3.

Bacteria, with their short generation times, serve as ideal starting points for investigations into the structure-activity relationships of ionic liquids (ILs). In this study, the following [TEA][AA] salts demonstrated inhibitory effects against *S. aureus*: [TEA][Lys], [TEA][Arg], TEA, [TEA][Ala], [TEA][Pro], [TEA][Ser], [TEA][Asp], and [TEA][Glu]. The strongest inhibitory properties against *S. aureus* were observed in [TEA][AA] salts with basic amino acid anions, specifically [TEA][Lys] and [TEA][Arg]. Their MIC values, 1.88% and 3.75%, respectively, were lower than that of the reference compound TEA (6.25%). The compounds [TEA][Ala], [TEA][Pro], and [TEA][Ser] were characterized by similar antimicrobial activity to pure TEA. On the other hand, [TEA][AA] salts with acidic amino acids in the structure showed the weakest inhibitory effect ([TEA][Asp] and [TEA][Glu] = 15%). Only [TEA][Arg] was able to determine an MBC = 3.75%.

IL effectively combating *E. coli* turned out to be basic [TEA][Lys], which at a concentration of 0.94% inhibited the growth of this bacteria. Other liquids with similar antibacterial properties to TEA (MIC = 3.12%) were alkaline [TEA][Arg] and non-polar [TEA][Ala], whose MIC was 3.75%. The remaining [TEA][AA] salts: [TEA][Thr], [TEA][Pro], [TEA][Ser], and acidic [TEA][Asp] and [TEA][Glu] were characterized by lower bacterial growth inhibiting capacity than TEA. Lower bactericidal properties of the tested liquids were observed compared to the reference compound, which was TEA (MBC = 3.13%). Liquids with anions of basic amino acids, namely [TEA][Lys] and [TEA][Arg] (MBC = 3.75%), had the strongest bactericidal effect, similar to TEA. On the other hand, [TEA][AA] salts based on acidic amino acids ([TEA][Asp] and [TEA][Glu]) showed the lowest bactericidal properties, MBC equal to 15%.

The trend of increasing antimicrobial activity for *C. albicans*, corresponding to the polarity of the side chain of amino acid anions, was as follows:

[TEA][Glu] ≈ [TEA][Asp] < [TEA][Pro] < TEA < [TEA][Met] < [TEA][Thr] » [TEA][Ser] < [TEA][His] < [TEA][Ala] < [TEA][Arg] » [TEA][Lys].

[TEA][Lys] and [TEA][Arg] (MIC = 0.94%) had the strongest inhibitory effect on yeast growth, and [TEA][Glu] and [TEA][Asp] (MIC = 15%) the weakest compared to pure TEA. It was also observed that [TEA][Asp] showed a stronger bactericidal effect (MBC = 15%) than TEA (MBC = 25%). An interesting observation is that more of the tested [TEA][AA] salts produced an inhibitory effect against *C. albicans* than against *S. aureus* and *E. coli*. In addition, [TEA][AA] salts have a greater bactericidal efficacy against *E. coli* than *S. aureus*. Hence, it may be concluded that [TEA][AA] salts are more likely to have antifungal than antibacterial properties.

The results obtained by us, as well as the research conducted by Wu et al. [31], who studied series of ionic liquids based on imidazolium and choline cations with different amino acids and anions, confirm that ionic liquids with the Asp anion are characterized by the lowest toxicity towards the tested microorganisms. This was due to having one hydrophilic carboxyl group in the IL, which is not easy to pass through the membrane of microorganisms [31]. The toxic effects of ionic liquids (ILs) on microorganisms can vary significantly due to the distinct structures of their cell walls. In Gram-positive bacteria, the cell wall is characterized by a thick layer of peptidoglycan, which can account for up to 90% of its composition. This thick peptidoglycan layer is rich in polysaccharides and provides considerable rigidity and structural support (R0) [40]. Additionally, the cell wall contains teichoic acids, which are hydrophobic and can interact with ILs. This hydrophobic nature of the cell wall influences how ILs penetrate and affect these bacteria [41]. In contrast, gram-negative bacteria have a more complex cell wall structure. Their cell wall consists of a thinner peptidoglycan layer situated between an inner cytoplasmic membrane and an outer membrane. The outer membrane of gram-negative bacteria is composed of lipopolysaccharides (LPS), which form a protective barrier against various compounds, including ILs [42]. This outer membrane has hydrophobic properties and can limit the penetration of ILs, thereby affecting the bacterial response to these substances [43]. Yeasts, which are eukaryotic microorganisms, have a cell wall that differs from both gram-positive and gram-negative bacteria. The cell walls of these microbes are primarily composed of glucans (e.g., β-glucans) and mannans, which provide structural integrity and protection. The structure of the yeast cell wall also includes chitin, a polymer of N-acetylglucosamine, which contributes to its rigidity [44,45]. The different chemical and physical properties of yeast cell walls can influence how ILs interact with and affect these organisms. However, our study showed equal or lower MIC values when applying the same [TEA][AA] salts to gram-negative than gram-positive bacteria compared to studies by Wu et al. where they reported a higher level of tolerance of gram-negative than gram-positive bacteria. Yeasts were the most sensitive microorganisms to the toxic effects of the [TEA][AA] salts we studied, which is also consistent with the results obtained in the studies of Wu et al. [31]. The effect of the ionic liquid on bacterial cells is primarily associated with the disintegration of the cell wall. The action of ILs on bacteria is generally less specific but faster compared to standard antibiotics, which exert their effects by interfering with cell wall synthesis, inhibiting enzymes or nucleic acid synthesis, disrupting the bacterial membrane structure, or inhibiting metabolic pathways [46].

Our study found that gram-negative bacteria were more sensitive to the toxic effects of [TEA][AA] salts than gram-positive bacteria, aligning with Hou et al. [33]. This contradicts the general trend of gram-negative bacteria being more resistant to biocides. The toxicity of these salts varied depending on their functional groups. Those with hydroxyl groups exhibited toxicity similar to pure TEA, while salts containing carboxyl groups ([TEA][Asp] and [TEA][Glu]) were the least toxic [33,47]. In contrast, salts with basic amino acid anions showed high toxicity, a pattern also observed by Hou et al. The greater toxicity of [TEA][AA] salts compared to choline chloride can be attributed to TEA’s three 2-hydroxyethyl chains, in contrast to choline chloride’s single 2-hydroxyethyl chain. Earlier we reported the antibacterial activity of ILs based on choline amino acids ([Ch][Ser], [Ch][Val], [Ch][Pro], [Ch][His], [Ch][Ala]) against the gram-positive bacterium *L. monocytogenes* and the gram-negative bacteria *A. hydrophila* and *K. pneumoniae*; all ILs showed relatively similar EC_50_ toxicity to microorganisms except for the least performing [Ch][Ser] [24].

Another future of ILs, such as increasing lipophilicity by elongating the alkyl chains in the cation as well as in the anion, leads to an enhancement of the toxic effect on microorganisms. However, this has not been confirmed in our research [33,41]. However, the use of an appropriate cation may decrease the toxic effect. The use of the [OHBMIM] cation turned out to be less toxic than the [Cho] and [OMIM] cations with Ser and Pro anions against gram-negative bacteria *Aliivibrio fischeri*. In the case of [Cho][Ala], it was detected to be less toxic than [OHBMIM][Ala] and the most toxic to *A. fischeri* [48].

The salt of amino acid in combination with benzalkonium as a cation showed a variance in antimicrobial effect for *S. aureus*, *S. epidermidis*, *Pseudomonas aeruginosa*, *E. coli*, and *Raoultella ornithinolytica*. The highest inhibitory effect was observed for [BA][Tyr], [BA][Pro], and [BA][Ile] (MIC = 0.5 mg L^−1^) against *S. aureus*. However, these compounds had the weakest inhibitory and bactericidal activity against *S. epidermidis*. [BA][Met] and [BA][Tyr] (MIC = 0.5 mg L^−1^) action against *P. aeruginosa* were similar to benzalkonium chloride. *E. coli* was the most resistant strain to the tested compounds, except for [BA][Leu], which acted similarly to benzalkonium chloride (MIC = 15 mg L^−1^) [49].

The influence of ILs with amino acid anions of proline and histidine and based on bis-ammonium or bis-phosphonium cations with an alkyl linker or a linker containing two ester bonds on gram-positive bacteria: *S. aureus*, *S. epidermidis*, *Enterococcus faecalis*, *Bacillus subtilis*, *Micrococcus luteus*, *Clostridium perfringens*, *Lactiplantibacillus plantarum*, gram-negative bacteria: *E. coli*, *P. aeruginosa*, *Serratia marcescens*, *Proteus vulgaris*, *Moraxella catarrhalis*, *Salmonella enteritidis*, and fungi: *C. albicans*, *Rhodotorula mucilaginosa*, *Botrytis cinerea*, *Fusarium graminearum* was determined [50]. Most of the tested compounds (19 out of 24) showed no activity or slight inhibition of some of the tested microorganisms. ILs with quaternary phosphorus atoms were more effective in inhibiting microbial growth than those with quaternary nitrogen atoms. Salts containing in their structure an alkyl chain with 12 carbon atoms were characterized by the highest antimicrobial activity. The presence of ester bonds resulted in a decrease in antimicrobial properties, and ILs with two ester bonds in the quaternary ammonium cation were among the weakest compounds. Among the tested compounds, the strongest bacterial growth inhibitory effect was observed for C_12_-bis-ammonium prolinate, C_12_-1,ω-bis(carboxymethyltributylammonium) prolinate, C_8_-bis-phosphonium prolinate, C_12_-bis-phosphonium prolinate, and C_12_-bis-phosphonium histidinate, which was at a level similar to the comparative compounds: [DDA][Cl] and [BA][Cl]. The MIC ranged from <0.5 to 125 μg mL^−1^ for gram-positive bacteria, from <0.5 to > 1000 μg mL^−1^ for gram-negative bacteria, and from 8 to 1000 μg mL^−1^ for the tested fungi. In the conducted research, we observed that the choice of anion type has a strong influence on the antifungal properties. The [TEA][His] showed stronger inhibition than [TEA][Pro] against *C. albicans*. However, in research led by Kaczmarek et al. [50], the degree of inhibition against *C. albicans* was the same when the C_12_-bis-phosphonium cation was used with His and Pro anions.

The next developed IL, based on TEA with Ala, Pro, and Ser anions, exhibited the same level of toxicity against *S. aureus* compared to Ghanem et al. [25]. In contrast, [TEA][Ala] was more potent than [TEA][Pro] and [TEA][Ser] against *E. coli*, as was [C_2_mpyrr][Ala] in comparison to [C_2_mpyrr][Ser] and [C_2_mpyrr][Pro]. Ghanem et al. [51] also reported the antimicrobial activity (*Aeromonas hydrophila*, *E. coli*, *Listeria monocytogenes*, and *S. aureus*) of ILs based on the 1-ethyl-1-methylpyrrolidinium cation [C_2_mpyrr] with amino acid anions: Gly, Ala, Ser, and Pro. The EC_50_ toxicity grade was as follows: [C_2_mpyrr][Ser] < [C_2_mpyrr][Pro] < [C_2_mpyrr][Ala] < [C_2_mpyrr][Gly]. These associations were referenced to earlier work by Ghanem et al. [25], in which ILs with the cation 1-octyl-3-methylimidazolium [OMIM] and 1-(2-hydroxyethyl)-3-methylimidazolium [C_2_OHMIM] with the above anions were studied. ILs with serinate anion showed the lowest toxicity compared to other compounds. [OMIM][AA] showed higher toxicity than [C_2_OHMIM][AA] and [C_2_mpyrr][AA] [51]. The last of the created based on the aromatic nonpolar amino acid IL, [TEA][Phe], does not cause any antimicrobial properties in our studies. Sivapragasam et al. [52] in studies indicated the high toxicity of ILs to the effective tetrabutylphosphonium [P_4444_] and tetrabutylammonium [N_4444_] cations with the Phe anion compared to those with the anion acetate [Ac] and taurinate [Tau] against *S. aureus* and *E. coli*. These findings highlight the crucial role of IL salt composition in determining antimicrobial activity, confirming the significance of interactions between IL components in shaping their potential antimicrobial effects.

## 3. Materials and Methods

### 3.1. Chemicals and Materials

Triethanolammonium salts of amino acids, [TEA][AA], used in the presented studies, were synthesized on our own by the reaction of triethanolammonium hydroxide and the respective amino acid. The synthesis method and identification of [TEA][AA] were described by us in an earlier article [26] and were briefly mentioned in the Supplementary Materials.

In the toxicity tests, the cress seeds *Lepidium sativum* L. purchased from a commercial shop PlantiCo Zielonki (Stare Babice, Poland) and mouse fibroblast cells (L929, ATCC CCL-1) purchased from Sigma Aldrich (Warsaw, Poland) were used as bioindicators. In antimicrobial tests, the bacteria *Escherichia coli* ATCC 8739 and *Staphylococcus aureus* ATCC 6538 as well as yeast *Candida albicans* ATCC 10231 were used. Microorganisms were taken from the collection of the Department of Microbiology and Biotechnology of the West Pomeranian University of Technology in Szczecin (Poland). All strains were stored at −80 °C in glycerol stocks before use.

Cell culture reagents such as Dulbecco’s Modified Eagle Medium (DMEM), L-glutamine, fetal bovine serum (FBS), Trypsin, PBS, Tween, resazurin penicillin, and streptomycin were purchased from Sigma Aldrich (Warsaw, Poland). Plastic cell culture dishes were purchased from VWR International Sp. z o. o. (Gdańsk, Poland). MHB (Mueller Hinton Broth) and MHA (Mueller Hinton Agar) were purchased from Biomaxima S.A. (Lublin, Poland).

### 3.2. Phytotoxicity Tests of [TEA][AA] Salts

The determination of the effect of [TEA][AA] salts on the seed germination and root growth of higher plants was carried out analogously to the methodology described in the standard ISO 18763:2016 [28]. The assays were performed with the seeds of *Lepidium sativum* L. as representative of dicotyl plant species. In contrast to the ISO standard, in our studies we did not use soil as a substrate. Instead, we used Petri dishes in which we placed polypropylene disks moistened with the tested solution, and on them we sowed cress seeds. The exact methodology for performing phytotoxicity tests is presented in Section 3.3 and Section 3.4.

### 3.3. Evaluation of Cress Seed Germination Degree

Twenty seeds of *Lepidium sativum* L. were placed in a Petri dish (diameter 5.5 cm) with the bottom covered by a perforated polypropylene disk against a paper filter to prevent the absorption of [TEA][AA] salts. The setup was moistened with 4 mL of deionized water for the control sample or 4 mL of an aqueous solution of test compound at the following concentrations: 0.01%, 0.03%, 0.05%, 0.10%, 0.30%, 0.50%, 1.00%, 2.00%, 3.00%, 4.00%, and 5.00% (*w*/*v*) for the test sample. The Petri dishes were incubated at 25 °C in the dark for 24 h. After this period, the number of germinated seeds was counted. The assay was performed in triplicate for each compound concentration. Phytotoxicity was expressed as the inhibition of the cress seeds germination relative to the control sample and was calculated using the formula:

(1) $$\%I_{SG}=\frac{100\cdot (E_{k}-E_{t})}{E_{k}}$$

E_k_—the number of germinated seeds in the control sample 

E_t_—the number of germinated seeds in the test sample 

### 3.4. Evaluation of Cress Root Growth

A perforated plastic disk was placed in a Petri dish (diameter 12 cm), and cress seeds were sown. The setup was moistened with deionized water and placed in an incubator at 25 °C in the dark for 24 h. After this period, 20 sprouted seeds with roots not exceeding 1 mm in length were selected and placed on a perforated disk in a Petri dish (diameter 5.5 cm). The seeds were then moistened with deionized water for the control sample and with aqueous solutions of the test compound at the previously specified concentrations for the test samples. Then the Petri dishes were placed in an incubator at 25 °C in the dark for 24 h. After this time, the length of the cress root was measured. The inhibition of the cress root growth was calculated relative to the control sample using the following formula:

(2) $$\%I_{RG}=\frac{100\cdot (L_{k}-L_{t})}{L_{k}}$$

L_k—_average root length in the control sample [mm] 

L_t_—average root length in the test sample [mm] 

### 3.5. Mammalian Cell Culture Cytotoxicity Screening

The cytotoxicity effect of [TEA][AA] was screened in cell culture using the murine fibroblast cell line (L929, ATCC CCL-1) based on the NCI-60 panel (National Cancer Institute) [29]. Cells (passages 8–25) were maintained in growth media, consisting of Dulbecco’s Modified Eagle Medium (DMEM) containing 10% fetal bovine serum (FBS), 2 mM L-glutamine, 100 U mL^−1^ penicillin, and 100 µg mL^−1^ streptomycin, in a T25 flask. For the experiments, a sub-confluent T25 flask of L929 cells was trypsinized, and two 96-well plates were prepared: one seeded with 10,000 cells per well and the second seeded with 5000 cells per well. The plates were then incubated for 24 h to allow the cells to adhere and spread, after which the media was aspirated and replaced with 100 µL of growth media containing 5% *v*/*v* [TEA][AA] salts and serial, 3-fold dilutions spanning a range of <0.001% to 5%, with 5 technical replicates. For the plate seeded with 10,000 cells per well, the plate was incubated for 24 h, and then cell viability was assessed using an inverted light microscope (Delta Optical IB-100, Delta Optical, Mińsk Mazowiecki, Poland) via resazurin viability [53] using a fluorescent plate reader (excitation 540 nm, emission 590 nm, Biotek Synergy HTX, Biotek® Instruments, Inc., Winooski, VT, USA). For the plate seeded with 5000 cells per well, the same assessments were performed after 48 h of culture. Dose response data were analyzed in R software (RStudio v4.2.2.) using the drc package [54]. Models were selected using Akaike’s information criterion. 

### 3.6. Antimicrobial Activity Tests of [TEA][AA]

To determine MIC, doubling serial dilutions of [TEA][AA] in MHB (Mueller Hinton Broth) were set up in 96-well microtiter plates, starting with 100 μL IL and 100 μL inoculum. Positive controls consisted of 100 μL starting with 100 μL IL and 100 μL inoculum. Positive controls consisted of 100 μL inoculum and 100 μL MHB, while negative controls consisted of 200 μL MHB only. Each set of tests and controls was set up with three replicates. The microtiter plate was incubated for 24 h at 37 °C. After incubation, the optical density of the microbial growth was determined at 600 nm using a microplate reader, Tecan Infinite m200pro (Tecan, Grödig, Austria). The results were expressed as the average of three replicates. The MIC value was defined as the concentration of the compound that inhibited the growth of the microorganism by at least 90%. Following MIC determination, the MBC was determined by transferring 20 μL from each well that displayed no growth signs onto MHA (Mueller Hinton Agar) plates. MHA plates were then incubated in a stationary incubator at 37 °C overnight. 

### 3.7. Statistical Analysis of Experimental Data

Statistical analyses were performed using Statistica 14.0 software (TIBCO Software Inc., Palo Alto, CA, USA). Mean values were compared using one-way ANOVA, and the significance of differences between groups was assessed with Tukey’s post-hoc test. A *p*-value < 0.05 was considered statistically significant. Detailed statistical results, including group comparisons and significance levels, were provided in the Supplementary Materials.

## 4. Conclusions

Based on the obtained results, it can be concluded that amino acid ionic liquids based on the triethanolammonium cation can be considered as chemical compounds of low toxicity to terrestrial higher plants. Cress is more sensitive to the effects of [TEA][AA] on root growth than seed germination. Considering the series of [TEA][AA] according to the highest concentration, which did not inhibit the seed germination and the root growth, [TEA][Asp] salt was characterized by the lowest phytotoxicity. The other salts with decreasing toxicity can be ranked for both tests as follows: [TEA][Met], [TEA][Ala], and [TEA][Ser]. The most negative effect on the viability of mouse fibroblasts after 24 h was observed after exposure to [TEA][Met], and the lowest degree of cytotoxicity was for [TEA][Phe]. The acidic anion [TEA][Asp] was less toxic than the basic [TEA][Lys]. [TEA][AA] salts with basic amino acid anions show a stronger inhibitory effect than [TEA][AA] salts based on acidic amino acid anions. [TEA][AA] salts show a stronger inhibitory effect on the growth of yeast than bacteria. In addition, [TEA][AA] salts have a greater bactericidal efficacy against *E. coli* than *S. aureus*. Excessive and often unjustified administration of antibiotics contributes to the evolution of multidrug-resistant microorganisms. Studies of the antimicrobial properties of [TEA][AA] salts prove to be helpful in combating the resistance of pathogens to antibiotics. Our research can be used as a basis for further toxicity tests of higher organisms as well as more complex systems using ecological [TEA][AA] salts. Moreover, further research is needed not only to study the effect of anion on living organisms but also to broaden the knowledge about the effects of various [TEA][AA] salts (not only common ILs based on imidazole and ammonium), about which there are few articles. Therefore, it is important to perform cytotoxicity studies against different cell lines, defining a window of concentrations that are safe to use. 

The observed differences in the relative toxicity of TEA alone versus TEA + AA salts arise from the fundamental biological and structural diversity of the tested organisms. In mammalian cells and microorganisms, the beneficial modulatory effect of amino acid anions is clearly evident, likely due to their impact on membrane interactions, buffering effects, and reduced uptake. In plants, particularly in seed germination and root elongation assays, the response is inherently more complex due to factors such as the presence of a seed coat, variable absorption, and plant-specific detoxification pathways. These biological factors may mask or modulate the protective effects of amino acid anions observed in simpler cell systems.

## Figures and Tables

**Table 1:** Classification of [TEA][AA] salts based on no observed effects concentration (NOEC^SG^) for cress seed germination inhibition.

NOEC^SG^				
0.05%	0.10%	0.30%	0.50%	2.00%
[TEA][Ser]	[TEA][Ile]	[TEA][Met]	[TEA][Lys]	[TEA][Asp]
[TEA][Gln]	[TEA][Val]	[TEA][Ala]		
[TEA]_2_[Glu]	[TEA][Arg]			

**Table 2:** Classification of [TEA][AA] salts based on no observed effects concentration (NOEC^RG^) for cress root length growth.

NOEC^RG^			
0.01%	0.03%	0.05%	0.10%
[TEA][Leu]	[TEA][Ala]	[TEA][Met]	[TEA][Ser]
[TEA][Pro]	[TEA][Glu]		[TEA][Asp]
[TEA][Thr]			

**Table 3:** The MIC and MBC values for a range of [TEA][AA] salts against *S. aureus*, *E. coli*, and *C. albicans*.

[TEA][AA]	Bacterial Strains					
	*S. aureus* ATCC 6538		*E. coli* ATCC 8739		*C. albicans* ATCC 10231	
	MIC (%)	MBC (%)	MIC (%)	MBC (%)	MIC (%)	MBC (%)
[TEA][Ala]	7.50	nd	3.75	7.50	1.88	nd
[TEA][Thr]	nd	nd	7.50	nd	3.75	nd
[TEA][Pro]	7.50	nd	7.50	7.50	7.50	nd
[TEA][Met]	nd	nd	nd	nd	5.00	nd
[TEA][His]	nd	nd	nd	nd	2.50	nd
[TEA][Phe]	nd	nd	nd	nd	nd	nd
[TEA][Leu]	nd	nd	nd	nd	nd	nd
[TEA][Ser]	7.50	nd	7.50	7.50	3.75	nd
[TEA][Arg]	3.75	3.75	3.75	3.75	0.94	nd
[TEA][Asp]	15.00	nd	7.50	15.00	15.00	15.00
[TEA]_2_[Asp]	nd	nd	nd	nd	nd	nd
[TEA][Lys]	1.88	nd	0.94	3.75	0.94	nd
[TEA][Glu]	15.00	nd	7.50	15.00	15.00	nd
TEA	6.25	nd	3.13	3.13	6.25	25.00

MIC—minimum inhibitory concentrations; MBC—minimum bactericidal concentrations; nd—not detected.

## Data Availability

The data presented in this study are available in the Supplementary Materials.

## Supplementary Materials

The following supporting information can be downloaded at https://www.mdpi.com/article/10.3390/molecules30081712/s1, Table S1: Inhibition of the *Lepidium sativum* seeds germination (I_SG_, %) after 24 h incubation with different types and concentrations of compounds in the medium; Table S2: Inhibition of the root growth in the preliminary pregerminated *Lepidium sativum* germination (I_RG_, %) after 24 h incubation with different types and concentrations of compounds in the medium; Table S3: Effective dose of compounds at two different exposure times on the murine L929 fibroblasts; Table S4: The dose-dependent effect of TEA and its salts with amino acids on root growth (%I_RG_). The indexes (a, b, c) next to the means indicate no significant differences between the compared values at *p* > 0.05 (Tukey test); Table S5: The dose-dependent effect of TEA and its salts with polar amino acids on root growth (%I_RG_). The indexes (a, b, c) next to the means indicate no significant differences between the compared values at *p* > 0.05 (Tukey test); Table S6: The dose-dependent effect of TEA and its salts with basic amino acids on root growth (%I_RG_). The indexes (a, b, c) next to the means indicate no significant differences between the compared values at *p* > 0.05 (Tukey test); Table S7: The dose-dependent effect of TEA and its salts with non-polar amino acids on root growth (%I_RG_). The indexes (a, b, c) next to the means indicate no significant differences between the compared values at *p* > 0.05 (Tukey test); Figure S1: The photo of Petri dishes in the bioassay of cress seeds germination in the presence of different concentration of TEA in the medium; Figure S2: The photo of Petri dishes in the bioassay of cress seeds germination in the presence of different concentration of [TEA][Asp] in the medium; Figure S3: The photo of Petri dishes in the bioassay of cress seeds germination in the presence of different concentration of [TEA][Ala] in the medium; Figure S4: The photo of Petri dishes in the bioassay of cress root growth in the presence of different concentration of TEA in the medium; Figure S5: The photo of Petri dishes in the bioassay of cress root growth in the presence of different concentration of [TEA][Asp] in the medium; Figure S6: The photo of Petri dishes in the bioassay of cress root growth in the presence of different concentration of [TEA][Arg] in the medium.
