# Supplementary material for: Phytotoxicity, Cytotoxicity, and Antimicrobial Activity of Triethanolammonium Amino Acids Salts
Source: Molecules. 2025 Apr 11;30(8):1712. doi: 10.3390/molecules30081712 (PMC12029836; doi:10.3390/molecules30081712)
Supplement: Supplementary file 1 [file molecules-30-01712-s001.zip › molecules-3524951-supplementary.pdf]

Supplementary Materials

Phytotoxicity, Cytotoxicity, and Antimicrobial Activity of Triethanolammonium Amino Acids Salts

Barbara Hanna Roman [1,†], Magdalena Charęza [2], Radosław Drozd [2], Martyna Sokołowska [3], Peter Sobolewski [3,‡] and Ewa Janus [1,*]

[1] Department of Organic Chemical Technology and Polymer Materials, Faculty of Chemical Technology and Engineering, West Pomeranian University of Technology in Szczecin, Pułaskiego 10, 70-322 Szczecin, Poland; roman.barbara@zut.edu.pl or barbara.roman@pum.edu.pl
[2] Department of Microbiology and Biotechnology, Faculty of Biotechnology and Animal Husbandry, West Pomeranian University of Technology in Szczecin, Piastów 45, 70-311 Szczecin, Poland; magdalena.szymanska@zut.edu.pl (M.C.); radoslaw.drozd@zut.edu.pl (R.D.)
[3] Department of Polymer and Biomaterials Engineering, Faculty of Chemical Technology and Engineering, West Pomeranian University of Technology in Szczecin, Piastów 45, 70-311 Szczecin, Poland; martyna.sokolowska@zut.edu.pl (M.S.); pete.sd@gmail.com (P.S.)
* Correspondence: ejanus@zut.edu.pl; Tel.: +48-91-449-45-84

Table of contents:

1. Synthesis of triethanolammonium amino acids salts [TEA][AA]…………. S3
2. Phytotoxic assessment of TEA and [TEA][AA] based on two bioassays using *Lepidium sativum* seeds……………………………………………… S3-S6
3. Cytotoxic effect of TEA and [TEA][AA] on the murine L929 fibroblasts….. S7
4. The statistical analysis results for $\%I_{RG}$ S7-S9

1. Synthesis of triethanolammonium amino acids salts [TEA][AA]

The synthesis, identification and characteristic of physical properties of triethanolammonium amino acids salts [TEA][AA] were previously reported [1].

Briefly, the synthesis was as follows: Equimolar amounts of L-amino acid (AA) and triethanolamine (TEA) were weighed into a 100 mL round-bottom flask equipped with a magnetic stirring bar and dissolved in deionized water (30 mL of water per each 0.1 mol of amino acid). The solution was stirred for 12 h at ambient temperature and then the water was distilled off on a rotary evaporator under reduced pressure. Then the obtained [TEA][AA] was dried for 12 h at temperature of 50 °C, and the pressure 5 mbar in a vacuum dryer. The obtained compound was stored in a freezer.

2. Phytotoxic assessment of TEA and [TEA][AA] based on two bioassays using *Lepidium sativum* seeds

Table S1. Inhibition of the *Lepidium sativum* seeds germination (I_{SG}, %**)** after 24h incubation with different types and concentrations of compounds in the medium

	Concentration of compound, % (w/w)										
**Compound**	**0.01**	**0.03**	**0.05**	**0.10**	**0.30**	**0.50**	**1.00**	**2.00**	**3.00**	**4.00**	**5.00**
	**I_{SG}, %***										
**TEA**	1.7(±0.5)	3.4(±0.9)	5.2(±0.8)	6.9(±0.1)	8.6(±0.0)	10.2(±0.9)	11.9(±1.7)	15.3(±1.3)	23.7(±1.4)	42.4(±2.1)	55.9(±1.7)
**[TEA][Gly]**	0.0(±0.5)	0.0(±0.5)	1.7 (±1.2)	3.4 (±1.3)	6.9 (±0.8)	13.8(±2.6)	20.7(±3.1)	43.1(±1.6)	63.8(±2.2)	81.0(±1.7)	87.9(±0.9)
**[TEA][Ala]**	0.0(±1.2)	0.0(±0.8)	0.0(±1.2)	0.0(±0.8)	0.0(±0.5)	9.4(±1.4)	11.8(±1.6)	17.7(±0.8)	64.7(±0.8)	88.3(±1.3)	100.0(±0.5)
**[TEA][Val]**	0.0(±0.9)	0.0(±1.7)	0.0(±1.2)	0.0(±1.2)	1.9(±0.5)	3.8(±1.7)	5.7(±0.5)	17.0(±0.5)	28.3(±0.5)	34.0(±1.3)	52.8(±1.3)
**[TEA][Ile]**	0.0(±1.9)	0.0(±1.4)	0.0(±0.8)	0.0(±0.5)	3.6(±2.2)	5.4(±0.5)	17.9(±2.6)	23.2(±0.9)	26.8(±0,9)	39.3(±2.1)	80.4(±1.3)
**[TEA][Leu]**	0.0(±1.9)	0.0(±1.9)	5.7(±0.5)	6.6(±0.9)	7.5(±2.1)	11.3(±0.8)	15.1(±1.6)	32.1(±0.1)	58.5(±0.5)	84.9(±2.5)	100(±0.1)
**[TEA][Pro]**	3.7(±0.5)	6.5(±0.5)	9.3(±0.5)	16.7(±0.8)	18.5(±0.5)	20.4(±1.3)	25.9(±1.3)	29.6(±0.9)	40.7(±0.5)	51.9(±0.5)	79.6(±0.5)
**[TEA][Met]**	0.0(±0.2)	0.0(±0.1)	0.0(±0.1)	0.0(±0.1)	0.0(±0.0)	1.9(±1.3)	3.7(±0.5)	7.4(±0.9)	14.8(±0.8)	29.6(±0.8)	37.0(±2.5)
**[TEA][Thr]**	0.0(±0.0)	3.8(±0.1)	6.7(±0.8)	11.3(±1.7)	15.1(±0.8)	16.8(±0.9)	18.9(±1.7)	21.7(±0.8)	24.5(±0.9)	45.3(±2.1)	77.4(±1.4)
**[TEA][Ser]**	0.0(±0.0)	0.0(±0.0)	0.0(±0.0)	2.8(±1.7)	5.7(±1.3)	6.6(±0.9)	7.5(±1.7)	17.0(±0.5)	18.9(±2.4)	34.0(±2.6)	75.5(±0.5)
**[TEA][Arg]**	0.0(±0.0)	0.0(±0.0)	0.0(±0.0)	0.0(±0.0)	1.2(±0.8)	5.5(±0.5)	15.5(±0.5)	25.5(±2.1)	32.7(±1.7)	58.2(±1.2)	76.4(±0.9)
**[TEA][Lys]**	0.0(±0.0)	0.0(±0.0)	0.0(±0.0)	0.0(±0.0)	0.0(±0.0)	0.0(±0.0)	1.9(±0.8)	11.5(±1.9)	38.5(±1.2)	57.7(±3.1)	76.9(±0.8)
**[TEA][His]**	0.0(±0.0)	1.9(±0.5)	3.7(±0.9)	5.6(±0.5)	7.4(±0.5)	9.3(±0.5)	12.0(±1.4)	14.8(±0.9)	25.9(±1.2)	27.8(±2.8)	74.1(±0.5)
**[TEA][Phe]**	0.0(±0.0)	0.0(±0.0)	0.9(±0.8)	1.8(±0.9)	3.5(±1.7)	5.3(±0.8)	8.8(±1.3)	10.5(±0.8)	14.0(±0.5)	22.8(±2.4)	86.0(±2.1)
**[TEA][Trp]**	0.0(±0.0)	1.9(±0.5)	3.7(±1.3)	12.0(±0.8)	16.7(±2.2)	18.5(±1.8)	28.7(±1.3)	38.9(±1.6)	53.7(±1.7)	61.1(±1.4)	83.3(±0.8)
**[TEA][Asn]**	5.6(±2.9)	6.5(±1.3)	7.4(±0.5)	9.3(±1.3)	12.0(±0.9)	14.8(±2.4)	20.4(±2.6)	33.3(±2.9)	46.3(±0.9)	67.6(±2.9)	88.9(±1.4)
**[TEA][Gln]**	0.0(±0.0)	0.0(±0.0)	0.0(±0.0)	1.7(±2.2)	9.2(±1.3)	15.0(±0.5)	20.8(±2.6)	38.1(±2.1)	53.8(±2.5)	86.7(±1.3)	100(±0.0)
**[TEA][Asp]**	0.0(±0.0)	0.0(±0.0)	0.0(±0.0)	0.0(±0.0)	0.0(±0.0)	0.0(±0.0)	0.0(±0.0)	0.0(±0.0)	3.5(±2.1)	16.2(±1.3)	28.8(±1.9)
**$[TEA]_2[Asp]$**	1.8(±0.1)	3.6(±1.3)	7.3(±0.8)	9.1(±0.5)	12.7(±0.8)	16.4(±1.3)	18.2(±0.8)	19.1(±1.3)	20.0(±0.5)	30.9(±0.9)	50.9(±0.8)
**[TEA][Glu]**	0.0(±0.0)	0.0(±0.0)	1.8(±0.5)	3.6(±0.8)	5.4(±1.3)	8.9(±0.8)	10.7(±1.3)	10.7(±1.3)	14.3(±0.8)	21.4(±0.5)	33.9(±2.6)
**$[TEA]_2[Glu]$**	0.0(±0.0)	0.0(±0.0)	0.0(±0.0)	3.7(±0.9)	5.6(±0.9)	7.4(±0.9)	14.8(±2.5)	22.2(±2.2)	29.6(±1.7)	37.0(±0.5)	63.0(±0.5)

* $I_{SG} = 100 (E_k - E_t) / E_k$, where E_k –the number of germinated seeds in the control sample, E_t – the number of germinated seeds in the test sample; mean(±SD), n = 3;

Table S2. The inhibition of the root growth in the preliminary pregerminated *Lepidium sativum* seeds (%I_{RG}**)** after 24h incubation with different types and concentrations of compounds in the medium

	Concentration of compound, % (w/w)										
**Compound**	**0.01**	**0.03**	**0.05**	**0.10**	**0.30**	**0.50**	**1.00**	**2.00**	**3.00**	**4.00**	**5.00**
	I_{RG}, %										
**TEA**	1.3(±1.5)	3.4(±2.3)	5.4(±1.9)	9.1(±1.8)	12.9(±1.9)	49.7(±1.8)	56.6(±1.2)	76.1(±0.8)	83.5(±0.7)	87.2(±0.9)	88.9(±0.7)
**[TEA][Gly]**	8.7(±1.8)	16.2(±1.8)	20.9(±2.0)	20.6(±1.7)	49.3(±1.3)	62.3(±1.3)	75.7(±0.7)	80.6(±0.6)	93.3(±0.5)	96.2(±0.4)	95.9(±0.4)
**[TEA][Ala]**	0.0(±0.0)	0.0(±0.0)	14.8(±1.5)	19.7(±1.4)	27.9(±1.2)	36.1(±1.2)	55.2(±0.8)	67.2(±1.0)	77.6(±0.6)	79.3(±0.6)	89.7(±0.5)
**[TEA][Val]**	11.6(±3.3)	11.9(±3.3)	21.7(±2.8)	32.7(±2.9)	49.1(±2.5)	54.8(±2.6)	62.9(±2.4)	73.3(±1.5)	77.6(±1.7)	82.0(±1.0)	89.9(±0.74)
**[TEA][Ile]**	2.9(±1.7)	27.8(±1.4)	31.4(±1.9)	35.1(±1.5)	34.6(±1.6)	36.9(±1.6)	56.9(±1.2)	70.9(±1.0)	72.5(±0.7)	77.5(±0.6)	80.4(±0.3)
**[TEA][Leu]**	0.0(±0.0)	8.9(±2.5)	10.5(±2.1)	20.3(±1.7)	30.0(±1.9)	39.9(±1.5)	53.0(±1.0)	67.9(±0.9)	71.6(±0.8)	88.7(±0.7)	94.7(±0.5)
**[TEA][Pro]**	0.0(±0.0)	20.7(±2.1)	27.3(±2.2)	39.5(±1.9)	57.5(±1.5)	67.7(±1.1)	74.5(±1.2)	83.0(±0.7)	88.2(±0.6)	90.1(±0.8)	92.0(±0.7)
**[TEA][Met]**	0.0(±0.0)	0.0(±0.0)	0.0(±0.0)	1.9(±3.4)	22.8(±3.3)	30.1(±3.8)	52.1(±2.2)	60.7(±2.0)	64.9(±1.4)	77.4(±1.3)	77.8(±0.9)
**[TEA][Thr]**	0.0(±0.0)	17.7(±2.3)	19.7(±2.4)	21.8(±1.9)	37.8(±1.5)	66.6(±1.3)	73.7(±1.0)	80.3(±0.9)	88.0(±0.8)	92.7(±0.6)	96.2(±0.5)
**[TEA][Ser]**	0.0(±0.0)	0.0(±0.0)	0.0(±0.0)	0.0(±0.0)	14.6(±1.0)	25.3(±0.8)	70.2(±0.7)	77.8(±0.6)	79.5(±0.6)	81.3(±0.5)	91.2(±0.4)
**[TEA][Arg]**	9.0(±1.9)	20.6(±1.8)	31.2(±1.9)	41.9(±1.3)	53.8(±1.3)	56.5(±1.4)	65.4(±1.0)	74.4(±0.7)	84.7(±0.6)	93.7(±0.5)	98.3(±0.3)
**[TEA][Lys]**	9.2(±1.9)	14.6(±2.1)	20.0(±1.9)	24.5(±1.9)	52.7(±1.7)	62.3(±1.2)	63.5(±0.9)	78.9(±0.8)	84.9(±0.8)	90.2(±0.6)	94.9(±0.5)
**[TEA][His]**	1.1(±2.2)	12.9(±2.1)	15.7(±2.2)	22.1(±2.1)	28.6(±2.3)	37.1(±2.0)	48.6(±1.6)	62.9(±1.2)	72.9(±0.9)	80.0(±0.8)	84.3(±0.7)
**[TEA][Phe]**	23.6(±2.4)	24.4(±2.0)	28.5(±2.0)	44.2(±1.6)	48.1(±1.7)	53.1(±1.3)	60.2(±1.4)	69.6(±1.1)	78.3(±0.9)	83.5(±0.9)	92.1(±0.6)
**[TEA][Trp]**	38.1(±1.3)	41.1(±1.2)	48.8(±1.0)	51.9(±1.0)	53.3(±0.4)	54.7(±1.0)	57.6(±0.8)	69.0(±0.8)	76.0(±0.7)	88.8(±0.6)	95.0(±0.4)
**[TEA][Asn]**	4.5(±1.6)	11.6(±1.6)	16.0(±1.6)	32.5(±1.7)	34.9(±1.4)	42.7(±1.5)	58.9(±1.1)	76.9(±1.0)	81.5(±0.9)	85(±0.8)	88.2(±0.7)
**[TEA][Gln]**	13.0(±2.0)	20.7(±1.8)	21.0(±2.2)	26.9(±1.8)	50.5(±1.7)	63.6(±1.3)	72.9(±0.9)	78.1(±1.0)	85.2(±0.9)	92.4(±0.5)	93.6(±0.5)
**[TEA][Asp]**	0.0(±0.0)	0.0(±0.0)	0.0(±0.0)	0.0(±0.0)	9.1(±1.7)	12.9(±1.7)	28.4(±2.0)	43.9(±1.3)	44.2(±1.0)	56.3(±1.0)	68.3(±0.8)
**[TEA]$_2$[Asp]**	7.7(±1.9)	8.6(±2.1)	11.6(±1.9)	29.6(±2.0)	32.6(±1.5)	36.6(±1.6)	40.7(±1.2)	65.4(±0.7)	76.7(±0.7)	82.2(±0.6)	86.2(±0.5)
**[TEA][Glu]**	0.0(±0.0)	0.0(±0.0)	4.0(±2.4)	4.9(±2.7)	5.8(±2.7)	21.0(±2.4)	36.3(±2.2)	44.5(±1.8)	61.4(±1.0)	79.4(±0.8)	82.4(±0.5)
**[TEA]$_2$[Glu]**	10.0(±2.9	18.1(±2.7)	21.3(±2.7)	24.8(±2.3)7	28.4(±2.42)	44.4(±2.2)	51.5(±2.1)	68.2(±1.3)	78.5(±0.6)	87.0(±0.4)	90.5(±0.7)

I_{RG} =100 (L_k – L_t) / L_k, where L_k – average root length in the control sample [mm], E_t – average root length in the test sample; mean(±SD), n = 3;

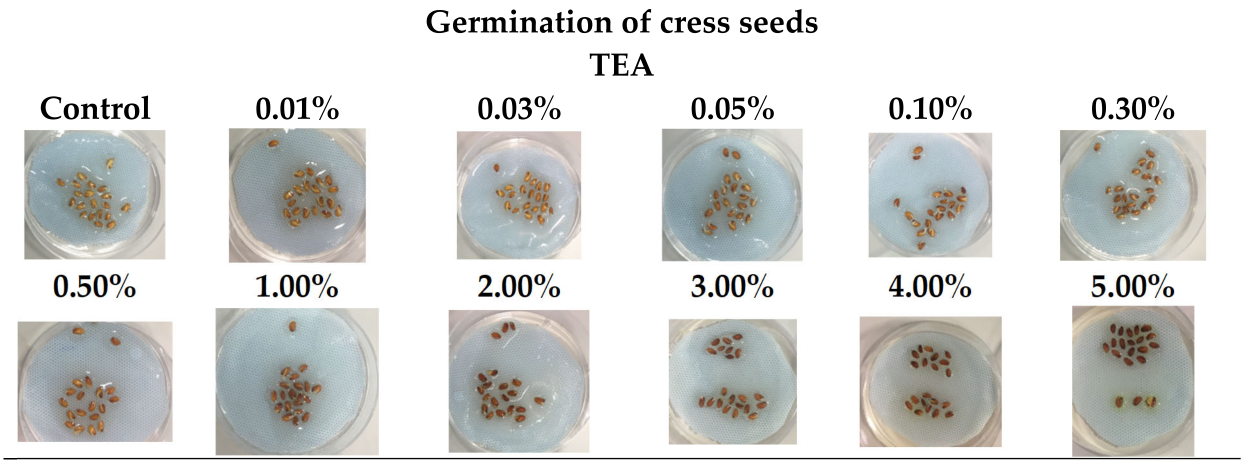

* at the top, seeds were separated that did not germinate

Figure S1. The photo of Petri dishes in the bioassay of cress seeds germination in the presence of different concentrations of TEA in the medium.

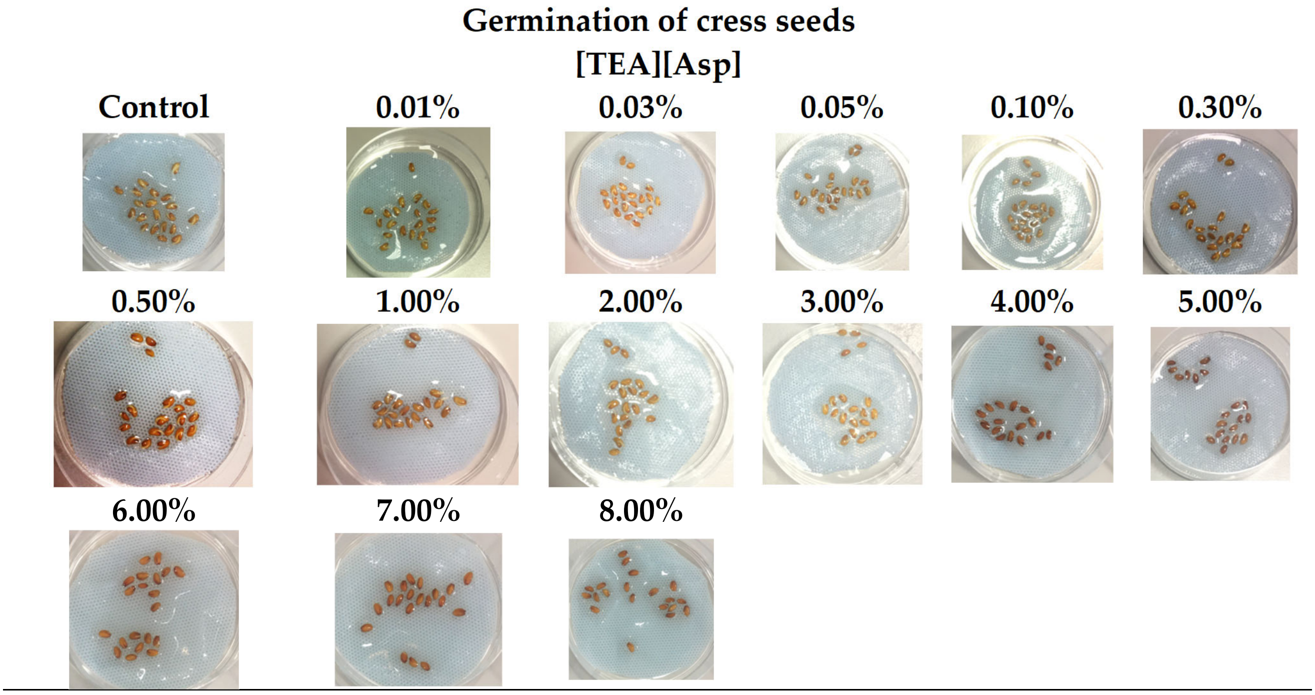

* at the top, seeds were separated that did not germinate

Figure S2. The photo of Petri dishes in the bioassay of cress seeds germination in the presence of different concentrations of [TEA][Asp] in the medium.

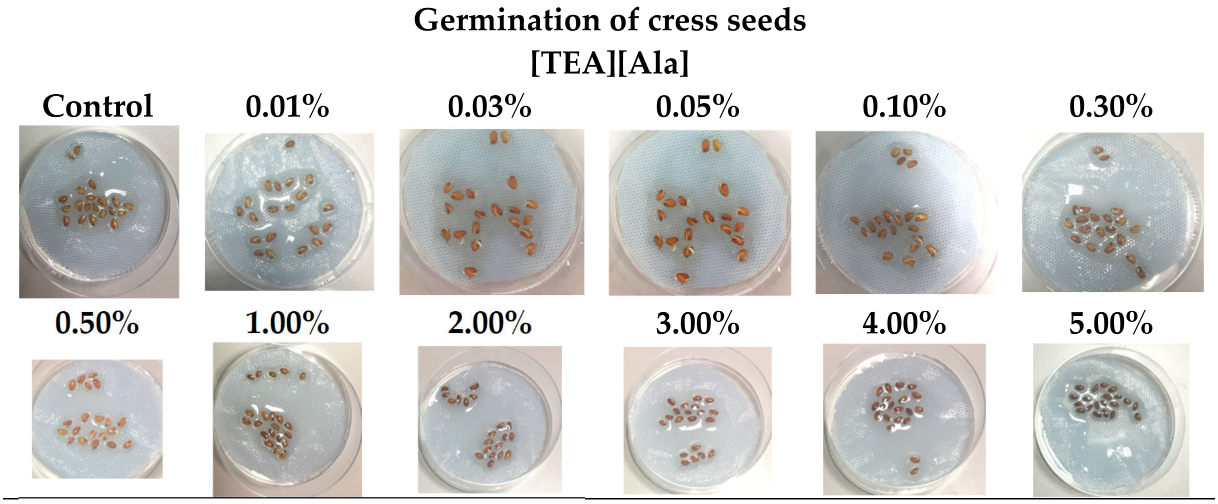

* at the top, seeds were separated that did not germinate

Figure S3. The photo of Petri dishes in the biossay of cress seeds germination in the presence of different concentrations of [TEA][Ala] in the medium.

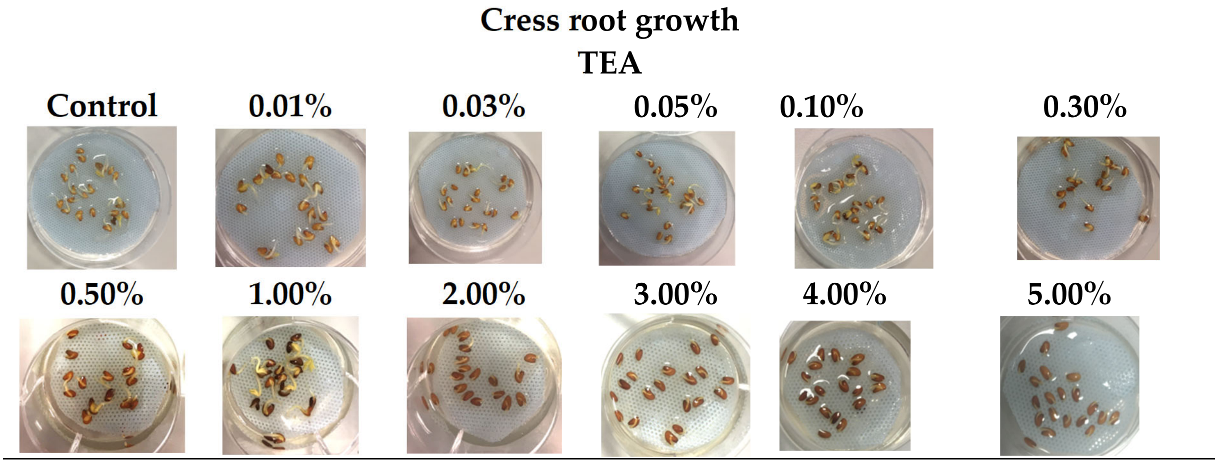

Figure S4. The photo of Petri dishes in the bioassay of cress root growth in the presence of different concentrations of TEA in the medium.

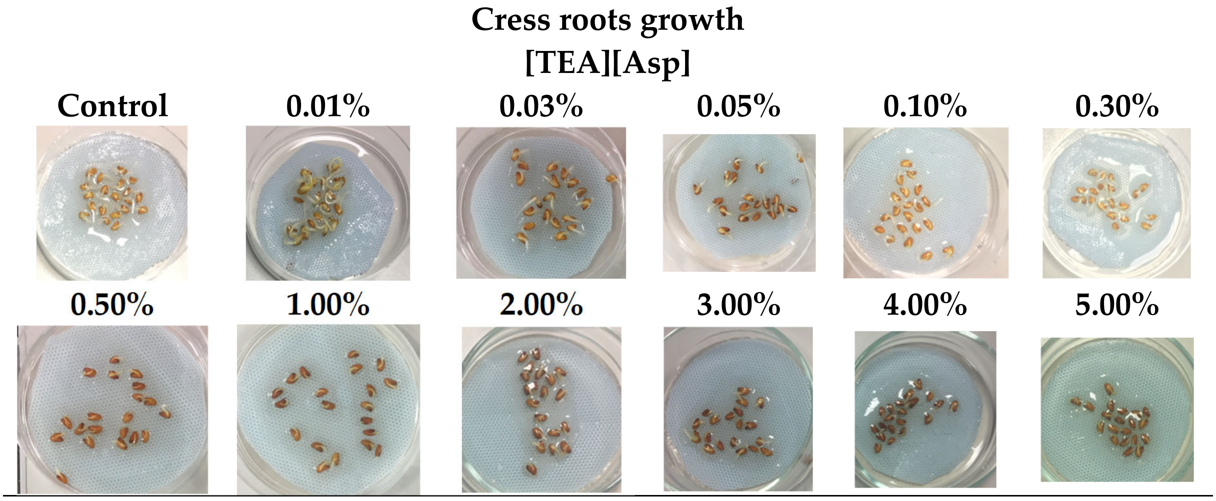

Figure S5. The photo of Petri dishes in the bioassay of cress roots growth in the presence of different concentrations of [TEA][Asp] in the medium.

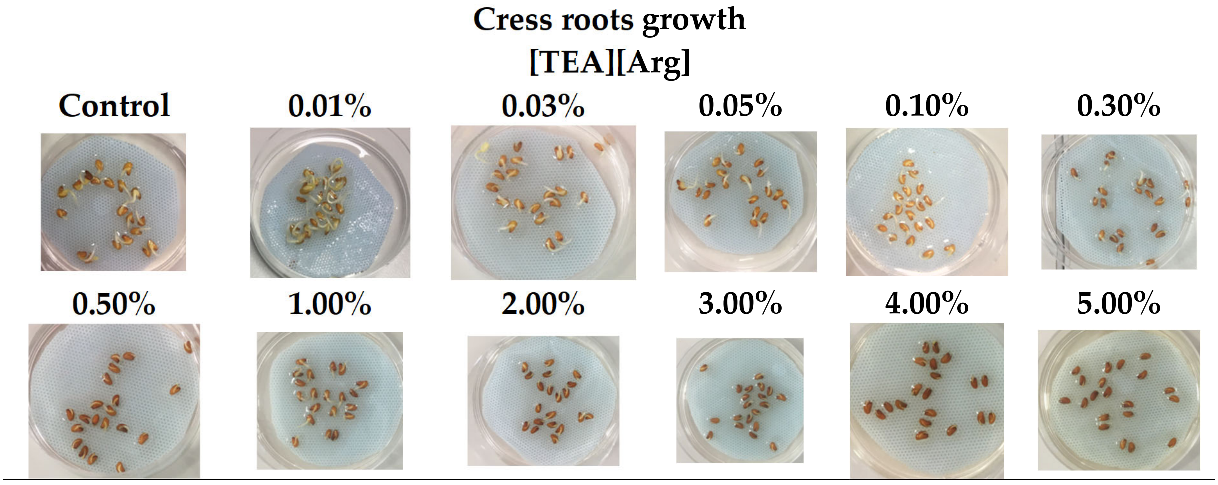

Figure S6. The photo of Petri dishes in the bioassay of cress roots growth in the presence of different concentrations of [TEA][Arg] in the medium.

3. Cytotoxic effect of TEA and [TEA][AA] on the murine L929 fibroblasts.

Table S3. Effective dose of compounds at two different exposure times on the murine L929 fibroblasts.

	24h			48h		
**Compound**	**ED$_{20}$[%]**	**ED$_{50}$[%]**	**ED$_{80}$[%]**	**ED$_{20}$[%]**	**ED$_{50}$[%]**	**ED$_{80}$[%]**
**TEA**	0.22±0.01	0.33±0.02	0.44±0.02	0.12±0.01	0.22±0.01	0.33±0.02
**[TEA][Leu]**	0.23±0.02	0.51±0.22	0.93±0.07	0.21±0.01	0.51±0.01	0.82±0.03
**[TEA][Met]**	0.08±0.01	0.29±0.02	0.74±0.08	0.09±0.01	0.31±0.02	0.78±0.06

**[TEA][Asp]**	0.47±0.03	0.66±0.03	0.86±0.09	0.30±0.02	0.50±0.01	0.71±0.01
**[TEA][Thr]**	0.40±0.09	0.57±0.03	0.73±0.14	0.26±0.03	0.38±0.02	0.50±0.03
**[TEA][Ser]**	0.26±0.03	0.55±0.04	0.98±0.11	np*	np*	np*
**[TEA][Ala]**	0.54±0.06	0.57±0.04	0.59±0.11	0.25±0.03	0.42±0.02	0.63±0.03
**[TEA][Pro]**	0.32±0.05	0.56±0.04	0.86±0.14	0.35±0.04	0.64±0.04	1.00±0.13
**[TEA][Lys]**	0.23±0.01	0.43±0.01	0.69±0.02	0.21±0.01	0.33±0.01	0.46±0.01
**[TEA][Phe]**	0.37±0.04	0.72±0.07	1.20±0.19	0.38±0.04	0.63±0.04	0.91±0.14

np* - not performed

4. The statistical analysis results for %I$_{RG}$

Table S4. The dose-dependent effect of TEA and its salts with acid amino acids on root growth (%I$_{RG}$). The indexes (a, b, c) next to the means indicate no significant differences between the compared values at p > 0.05 (Tukey test).

[TEA][AA](%)	[TEA]		[TEA][Asp]		[TEA]$_2$[Asp]		[TEA][Glu]		[TEA]$_2$[Glu]	
	Mean	SD*	Mean	SD	Mean	SD	Mean	SD	Mean	SD
0.01	1.3^{a}	1.49	0^{a}	0	7.7^{a}	1.91	0^{a}	0	10.0^{a}	2.91
0.03	3.4^{a}	2.29	0^{b}	0	8.6^{ab}	2.06	0^{b}	0	18.1^{a}	2.72
0.05	5.4^{a}	1.88	0^{a}	0	11.6^{a}	1.92	4.0^{a}	2.39	21.3^{a}	2.67
0.1	9.1a	1.83	0^{a}	0	29.6^{a}	2.02	4.9^{a}	2.67	24.8^{a}	2.37
0.3	12.9^{ab}	1.92	9.1^{ab}	1.74	32.6^{a}	1.48	5.8^{a}	2.68	28.4^{ab}	2.42
0.5	49.7^{a}	1.78	12.9^{b}	1.71	36.6^{abc}	1.63	21.0^{b}	2.37	44.4^{ac}	2.18
1	56.6^{a}	1.24	28.4	2.0	40.7^{ab}	1.16	36.3^{bc}	2.21	51.5^{ac}	2.11
2	76.1^{ab}	0.82	43.9^{c}	1.31	65.4^{ad}	0.73	44.5^{c}	1.84	68.2^{abd}	1.33
3	83.5^{a}	0.70	44.2^{b}	0.99	76.7^{a}	0.71	61.4^{b}	0.98	78.5^{a}	0.63
4	87.2^{a}	0.85	56.3	0.90	82.2^{a}	0.55	79.4^{a}	0.76	87.0^{a}	0.42
5	88.9^{a}	0.65	68.3	0.78	86.2^{a}	0.46	82.4^{a}	0.54	90.5^{a}	0.66

SD - standard deviations

Mean of n=3

Table S5. The dose-dependent effect of TEA and its salts with polar amino acids on root growth (%I$_{RG}$). The indexes (a, b, c) next to the means indicate no significant differences between the compared values at p > 0.05 (Tukey test).

[TEA][AA](%)	[TEA]		[TEA][Ser]		[TEA][Thr]		[TEA][Asn]		[TEA][Gln]	
	Mean	SD	Mean	SD	Mean	SD	Mean	SD	Mean	SD
0.01	1.3^{a}	1.49	0^{a}	0	0^{a}	0	4.5a	1.57	13.0^{a}	1.97
0.03	3.4^{a}	2.29	0^{b}	0	17.7^{a}	2.32	11.6^{a}	1.59	20.7^{a}	1.75
0.05	5.4^{a}	1.88	0^{b}	0	19.7^{c}	2.42	16.0^{ac}	1.58	21.0^{ac}	2.16
0.1	9.1^{a}	1.83	0^{b}	0	21.8^{c}	1.86	32.5^{c}	1.65	26.9^{a}	1.79
0.3	12.9^{a}	1.92	14.6^{ab}	1	37.8^{b}	1.51	34.9^{b}	1.42	50.5^{b}	1.65
0.5	49.7^{a}	1.78	25.3^{ab}	0.82	66.6^{b}	1.25	42.7^{a}	1.49	63.6^{ab}	1.25

1	56.6^{a}	1.24	70.2^{a}	0.72	73.7^{ab}	1.03	58.9^{a}	1.09	72.9^{ab}	0.86
2	76.1^{a}	0.82	77.8^{a}	0.59	80.3^{a}	0.94	76.9^{a}	0.98	78.1^{a}	1.03
3	83.5^{a}	0.70	79.5^{a}	0.58	88.0^{a}	0.81^{a}	81.5	0.86	85.2^{a}	0.92
4	87.2^{a}	0.85	81.3^{a}	0.47	92.7^{a}	0.61	85.0^{a}	0.75	92.4^{a}	0.51
5	88.9^{a}	0.65	91.2^{a}	0.43	96.2^{a}	0.45	88.2^{a}	0.71	93.6^{a}	0.48

SD - standard deviations

Mean of n=3

Table S6. The dose-dependent effect of TEA and its salts with basic amino acids on root growth (%I_{RG}). The indexes (a, b, c) next to the means indicate no significant differences between the compared values at $p > 0.05$ (Tukey test).

**[TEA][AA](%)**	**[TEA]**		**[TEA][His]**		**[TEA][Arg]**		**[TEA][Lys]**	
	Mean	SD	Mean	SD	Mean	SD	Mean	SD
0.01	1.3^{a}	1.49	9.2^{a}	1.92	9.0^{a}	1.88	1.1^{a}	2.18
0.03	3.4^{a}	2.29	14.6^{a}	2.10	20.6^{a}	1.83	12.9^{a}	2.10
0.05	5.4^{a}	1.88	20.0^{a}	1.93	31.2^{a}	1.85	15.7^{a}	2.16
0.1	9.1^{a}	1.83	24.5^{a}	1.90	41.9^{b}	1.33	22.1^{ab}	2.10
0.3	12.9^{a}	1.92	52.7^{a}	1.65	53.8^{ab}	1.34	28.6^{ab}	2.27
0.5	49.7^{a}	1.78	62.3^{a}	1.20	56.5^{a}	1.37	37.1^{a}	1.96
1	56.6^{a}	1.24	63.5^{ab}	0.92	65.4^{a}	1.00	48.6^{b}	1.58
2	76.1^{a}	0.82	78.9^{b}	0.82	74.4^{a}	0.68	62.9^{a}	1.15
3	83.5^{a}	0.70	84.9^{a}	0.78	84.7^{a}	0.62	72.9^{a}	0.90
4	87.2^{a}	0.85	90.2^{b}	0.60	93.7^{a}	0.46	80.0^{a}	0.83
5	88.9^{a}	0.65	94.9^{a}	0.46	98.3^{a}	0.26	84.3^{a}	0.70

SD - standard deviations

Mean of n=3

Table S7. The dose-dependent effect of TEA and its salts with non-polar amino acids on root growth (%I_{RG}). The indexes (a, b, c) next to the means indicate no significant differences between the compared values at $p > 0.05$ (Tukey test).

[TEA][AA]	[TEA]		[TEA][Gly]		[TEA][Ala]		[TEA][Leu]		[TEA][Ile]		[TEA][Val]		[TEA][PRO]		[TEA][Met]		[TEA][Phe]		[TEA][Trp]	
(%)	Mean	SD	Mean	SD	Mean	SD	Mean	SD	Mean	SD	Mean	SD	Mean	SD	Mean	SD	Mean	SD	Mean	SD
0.01	1.3^{a}	1.49	8.7^{a}	1.78	0^{a}	0	0^{a}	0	2.9^{a}	1.68	11.6^{a}	3.29	0^{a}	0	0^{a}	0	23.6^{a}	2.40	38.1^{b}	1.32
0.03	3.4^{a}	2.29	16.2^{a}	1.77	0^{ab}	0	8.9^{a}	2.50	27.8^{a}	1.41	11.9^{b}	3.31	20.7^{a}	2.08	0^{a}	0	24.4^{ab}	2.04	41.1^{b}	1.17
0.05	5.4^{a}	1.88	20.9^{ab}	2.03	19.7^{ab}	1.43	10.5^{a}	2.11	31.4^{c}	1.87	21.7^{ab}	2.75	27.3^{bc}	2.15	0^{a}	0	28.5^{bc}	1.99	48.8^{d}	0.92
0.1	9.1^{a}	1.83	20.6^{ab}	1.66	14.8^{a}	1.41	20.3^{a}	1.74	35.1^{c}	1.50	32.7^{bc}	2.92	39.5^{cd}	1.87	1.9^{a}	3.37	44.2^{d}	1.57	51.9^{d}	1.01
0.3	12.9^{a}	1.92	49.3^{bc}	1.29	27.9^{ab}	1.18	30.0^{ab}	1.92	34.6^{ab}	1.58	49.1^{bc}	2.52	57.5^{bc}	1.45	22.8^{ab}	3.28	48.1^{bc}	1.68	53.3^{c}	0.40
0.5	49.7^{ab}	1.78	62.3^{bc}	1.33	36.1^{ab}	1.15	39.9^{a}	1.51	36.9^{a}	1.62	54.8^{abc}	2.57	67.7^{c}	1.14	30.1^{a}	3.75	53.1^{abc}	1.34	54.7^{abc}	1.04
1	56.6^{ab}	1.24	75.7^{c}	0.72	55.2^{ab}	0.75	53.0^{a}	0.99	56.9^{ab}	1.18	62.9^{ab}	2.37	74.5^{bc}	1.22	52.1^{a}	2.21	60.2^{ab}	1.39	57.6^{ab}	0.80
2	76.1^{ab}	0.82	80.6^{ab}	0.57	67.2^{a}	0.95	67.9^{a}	0.88	70.9^{ab}	0.95	73.3^{ab}	1.48	83.0^{b}	0.65	60.7^{c}	1.99	69.6^{ab}	1.14	69.0^{ab}	0.83
3	83.5^{b}	0.70	93.3^{c}	0.49	77.6^{ab}	0.64	71.6^{a}	0.80	72.5^{a}	0.72	77.6^{a}	1.68	88.2^{bc}	0.58	64.9^{a}	1.40	78.3^{ab}	0.85	76.0^{ab}	0.72
4	87.2^{a}	0.85	96.2^{d}	0.41	79.3^{a}	0.63	88.7^{b}	0.73	77.5^{a}	0.60	82.0^{a}	1.04	90.1^{abc}	0.82	77.4^{a}	1.29	83.5^{ab}	0.85	88.8^{bc}	0.62
5	88.9^{a}	0.65	95.9^{c}	0.4	89.7^{b}	0.50	94.7^{c}	0.46	80.4^{a}	0.29	89.9^{b}	0.74	92.0b^{c}	0.70	77.8^{a}	0.93	92.1^{bc}	0.55	95.0^{c}	0.41

SD* - standard deviation

Mean of n=3

1. Roman, B.H.; Charęza, M.; Janus, E.; Drozd, R. Evaluation of New L-Amino Acids Triethanolammonium Salts Usability for Controlling Protease Activity. *Int. J. Biol. Macromol.* **2023**, *231*, 123218.
